# Genomic Anatomy of Homozygous XX Females and YY Males Reveals Early Evolutionary Trajectory of Sex-determining Gene and Sex Chromosomes in *Silurus* Fishes

**DOI:** 10.1093/molbev/msae169

**Published:** 2024-08-13

**Authors:** Tao Wang, Gaorui Gong, Zhi Li, Jun-Sheng Niu, Wen-Xuan Du, Zhong-Wei Wang, Yang Wang, Li Zhou, Xiao-Juan Zhang, Zong-Qiang Lian, Jie Mei, Jian-Fang Gui, Xi-Yin Li

**Affiliations:** Key Laboratory of Breeding Biotechnology and Sustainable Aquaculture (CAS), State Key Laboratory of Freshwater Ecology and Biotechnology, Hubei Hongshan Laboratory, The Innovative Academy of Seed Design, Institute of Hydrobiology, Chinese Academy of Sciences, Wuhan 430072, China; College of Advanced Agricultural Sciences, University of Chinese Academy of Sciences, Beijing 100049, China; College of Fisheries, Huazhong Agricultural University, Wuhan 430070, China; Key Laboratory of Breeding Biotechnology and Sustainable Aquaculture (CAS), State Key Laboratory of Freshwater Ecology and Biotechnology, Hubei Hongshan Laboratory, The Innovative Academy of Seed Design, Institute of Hydrobiology, Chinese Academy of Sciences, Wuhan 430072, China; College of Advanced Agricultural Sciences, University of Chinese Academy of Sciences, Beijing 100049, China; Key Laboratory of Breeding Biotechnology and Sustainable Aquaculture (CAS), State Key Laboratory of Freshwater Ecology and Biotechnology, Hubei Hongshan Laboratory, The Innovative Academy of Seed Design, Institute of Hydrobiology, Chinese Academy of Sciences, Wuhan 430072, China; Key Laboratory of Breeding Biotechnology and Sustainable Aquaculture (CAS), State Key Laboratory of Freshwater Ecology and Biotechnology, Hubei Hongshan Laboratory, The Innovative Academy of Seed Design, Institute of Hydrobiology, Chinese Academy of Sciences, Wuhan 430072, China; College of Advanced Agricultural Sciences, University of Chinese Academy of Sciences, Beijing 100049, China; Key Laboratory of Breeding Biotechnology and Sustainable Aquaculture (CAS), State Key Laboratory of Freshwater Ecology and Biotechnology, Hubei Hongshan Laboratory, The Innovative Academy of Seed Design, Institute of Hydrobiology, Chinese Academy of Sciences, Wuhan 430072, China; College of Advanced Agricultural Sciences, University of Chinese Academy of Sciences, Beijing 100049, China; Key Laboratory of Breeding Biotechnology and Sustainable Aquaculture (CAS), State Key Laboratory of Freshwater Ecology and Biotechnology, Hubei Hongshan Laboratory, The Innovative Academy of Seed Design, Institute of Hydrobiology, Chinese Academy of Sciences, Wuhan 430072, China; College of Advanced Agricultural Sciences, University of Chinese Academy of Sciences, Beijing 100049, China; Key Laboratory of Breeding Biotechnology and Sustainable Aquaculture (CAS), State Key Laboratory of Freshwater Ecology and Biotechnology, Hubei Hongshan Laboratory, The Innovative Academy of Seed Design, Institute of Hydrobiology, Chinese Academy of Sciences, Wuhan 430072, China; College of Advanced Agricultural Sciences, University of Chinese Academy of Sciences, Beijing 100049, China; Key Laboratory of Breeding Biotechnology and Sustainable Aquaculture (CAS), State Key Laboratory of Freshwater Ecology and Biotechnology, Hubei Hongshan Laboratory, The Innovative Academy of Seed Design, Institute of Hydrobiology, Chinese Academy of Sciences, Wuhan 430072, China; College of Advanced Agricultural Sciences, University of Chinese Academy of Sciences, Beijing 100049, China; Department of Fish Genetic Breeding, Ningxia Fisheries Research Institute, Yinchuan 750001, China; Key Laboratory of Breeding Biotechnology and Sustainable Aquaculture (CAS), State Key Laboratory of Freshwater Ecology and Biotechnology, Hubei Hongshan Laboratory, The Innovative Academy of Seed Design, Institute of Hydrobiology, Chinese Academy of Sciences, Wuhan 430072, China; College of Fisheries, Huazhong Agricultural University, Wuhan 430070, China; Key Laboratory of Breeding Biotechnology and Sustainable Aquaculture (CAS), State Key Laboratory of Freshwater Ecology and Biotechnology, Hubei Hongshan Laboratory, The Innovative Academy of Seed Design, Institute of Hydrobiology, Chinese Academy of Sciences, Wuhan 430072, China; College of Advanced Agricultural Sciences, University of Chinese Academy of Sciences, Beijing 100049, China; Key Laboratory of Breeding Biotechnology and Sustainable Aquaculture (CAS), State Key Laboratory of Freshwater Ecology and Biotechnology, Hubei Hongshan Laboratory, The Innovative Academy of Seed Design, Institute of Hydrobiology, Chinese Academy of Sciences, Wuhan 430072, China; College of Advanced Agricultural Sciences, University of Chinese Academy of Sciences, Beijing 100049, China

**Keywords:** sex determination, sex chromosome turnover, sex-determining gene, *amhr2y*, gene duplication, gene degeneration

## Abstract

Sex chromosomes display remarkable diversity and variability among vertebrates. Compared with research on the X/Y and Z/W chromosomes, which have long evolutionary histories in mammals and birds, studies on the sex chromosomes at early evolutionary stages are limited. Here, we precisely assembled the genomes of homozygous XX female and YY male Lanzhou catfish (*Silurus lanzhouensis*) derived from an artificial gynogenetic family and a self-fertilized family, respectively. Chromosome 24 (Chr24) was identified as the sex chromosome based on resequencing data. Comparative analysis of the X and Y chromosomes showed an approximate 320 kb Y-specific region with a Y-specific duplicate of anti-Mullerian hormone type II receptor (*amhr2y*), which is consistent with findings in 2 other *Silurus* species but on different chromosomes (Chr24 of *Silurus meridionalis* and Chr5 of *Silurus asotus*). Deficiency of *amhr2y* resulted in male-to-female sex reversal, indicating that *amhr2y* plays a male-determining role in *S. lanzhouensis*. Phylogenetic analysis and comparative genomics revealed that the common sex-determining gene *amhr2y* was initially translocated to Chr24 of the *Silurus* ancestor along with the expansion of transposable elements. Chr24 was maintained as the sex chromosome in *S. meridionalis* and *S. lanzhouensis*, whereas a sex-determining region transition triggered sex chromosome turnover from Chr24 to Chr5 in *S. asotus*. Additionally, gene duplication, translocation, and degeneration were observed in the Y-specific regions of *Silurus* species. These findings present a clear case for the early evolutionary trajectory of sex chromosomes, including sex-determining gene origin, repeat sequence expansion, gene gathering and degeneration in sex-determining region, and sex chromosome turnover.

## Introduction

Sex is considered to have a single evolutionary origin and to be present in the last common ancestor of eukaryotes (Speijer et al. 2015; Hofstatter and Lahr 2019), providing a vital driving force for the evolution of life due to the mixture of genetic material and meiotic recombination (Barton 2009; McDonald et al. 2016). Sex determination is the process that initiates the development of gonadal primordium (a group of cells that represents the initial stages of development of the gonads). This primordium is bipotential since it can develop into either an ovary or a testis. Females and males are determined by genotypic sex determination, environmental sex determination, or both (Capel 2017; Li and Gui 2018; Li et al. 2022). In species with genotypic sex determination, sex chromosomes typically originate from autosomes independently by acquiring sex-determining gene(s). Together with sexually antagonistic allele gathering, transposable element accumulation, chromosome inversion, and epigenetic changes around the sex-determining region, recombination is suppressed between proto-sex chromosomes, leading to heteromorphic sex chromosomes (Charlesworth and Charlesworth 2000; Furman et al. 2020). Apart from an independent origin, the diversity and variability of sex chromosomes are largely caused by frequent sex chromosome turnover, which usually involves the replacement of the ancestral sex chromosomes with a new set of sex chromosomes (Abbott et al. 2017). It is difficult to ascertain the early evolutionary trajectory of sex chromosomes in most studied mammals and birds, given that the origins of their sex chromosomes occurred over 100 million years ago (Mya) (Graves 2006; Zhou et al. 2014). Therefore, nonmodel animals, particularly fish species (Kitano et al. 2024), with more recently formed sex chromosomes represent an ideal system for elucidating the early evolution of sex chromosomes.

Sex-determining genes are derived from allelic diversification or gene duplication (commonly accompanied with gene translocation) and display diversity among vertebrates, particularly in fish species. For allele diversification, one of the alleles on autosomes evolves into a sex-determining gene through single nucleotide mutations (Koyama et al. 2019), indels (insertions and deletions) (Wang et al. 2022a), or changes in the regulatory region (Herpin et al. 2021). For gene duplication, a duplicated gene is inserted into either the ancestral chromosome or another chromosome and acquires the function of sex determination (Nakamoto et al. 2021; Pan et al. 2021a). Sex-determining genes usually arise from members of a sexual regulatory network, such as genes in the TGF-β (transforming growth factor beta) signaling pathway (Pan et al. 2021b), DM (doublesex and Mab-3) domain–containing genes (Matsuda et al. 2002), *sox* (*Sry*-related HMG box) family genes (Takehana et al. 2014), and genes in the steroid pathway (Koyama et al. 2019). Genes that do not regulate gonadal differentiation and development also have evolved to become sex-determining genes. For example, *sdy* (sex-determining gene on the Y chromosome) in rainbow trout (*Oncorhynchus mykiss*) is derived from an immune-related gene (Yano et al. 2012). Among all the sex-determining genes identified in vertebrates, genes from the TGF-β signaling pathway account for the majority (Pan et al. 2021b; Li et al. 2022). To determine why members of the TGF-β pathway are frequently recruited as master regulators and how their signaling is integrated with sex determination and differentiation, it is necessary to identify sex-determining genes across a wide range of taxa.

With more than 4,000 species, catfish (order: Siluriformes) have been widely used to study sex determination mechanisms and sex control breeding in aquaculture (Kappas et al. 2016; Gui et al. 2022; Zhou et al. 2023). Recently, *amhr2y* (Y-linked anti-Mullerian hormone type II receptor) was identified as a candidate sex-determining gene in southern catfish (*Silurus meridionalis*) and Amur catfish (*Silurus asotus*), but on different newly formed sex chromosomes (chromosome 24 [Chr24] of *S. meridionalis* and chromosome 5 [Chr5] of *S. asotus*) (Zheng et al. 2022, 2023). However, the evolutionary process that led to the different locations of *amhr2y* in *Silurus* species is still unclear. Lanzhou catfish (*Silurus lanzhouensis*) (also known as the Yellow River catfish) with wide distributions around the reaches of the Yellow River (Chen 1977) is closely related to *S. meridionalis* and *S. asotus* and also displays obvious sexual dimorphism in growth rate and body size (Niu et al. 2024). The identification of the sex chromosome and sex-determining gene in *S. lanzhouensis* will facilitate the illustration of sex determination system evolution and the implementation of sex control breeding in *Silurus* species (Gui 2024).

Separate assembly of X and Y chromosomes from an XY individual with homomorphic sex chromosomes is still an important challenge. Although current sequencing techniques can distinguish homozygous sex chromosomes (Huang et al. 2022; Akagi et al. 2023), there is still no way to ensure that the assembled sex chromosomes do not contain chimeric sequences. In comparison to mammals and birds, fish sex chromosomes are commonly less divergent and their sex is sensitive to environmental factors (Capel 2017). Consequently, multiple genotypes of individuals can be generated in some fish species, including XX, XY, and YY females and males (Gong et al. 2022). The genomic anatomy of XX and YY individuals with homozygous sex chromosomes can be employed to address the issue of chimeric sequences derived from the sequencing of XY individuals with heterozygous sex chromosomes.

In previous studies, the XX/XY sex determination system was identified in *S. lanzhouensis* (Wang et al. 2021). We established an artificial gynogenetic (development of the larvae containing only maternal genetic information due to the activation of the eggs with the irradiated sperm) family with all XX females by cold shock. Furthermore, an occasional hermaphrodite individual with both ovarian and testicular tissues was identified. A self-fertilized family was established using the eggs and sperm of the intersexual gonad (Wang et al. 2021). The hermaphrodite individual had an XY genotype, resulting in the production of XX females, XY males, and YY males in the self-fertilized family (Wang et al. 2021). The genetic homozygosity of both artificial gynogenetic offspring (72.87% homozygous and 27.13% heterozygous single-nucleotide polymorphism [SNP] loci) and self-fertilized offspring (73.02% homozygous and 26.98% heterozygous SNP loci) is significantly higher than that of normal sexually reproduced offspring (55.60% homozygous and 44.40% heterozygous SNP loci) (Niu et al. 2024). Therefore, we can use the ideal materials, the XX female from the artificial gynogenetic family and the YY male from the self-fertilized family, to precisely elucidate the X and Y chromosomes of *S. lanzhouensis* and to illustrate the evolution of sex determination system in *Silurus* species.

In this study, the genomes of the homozygous XX female and YY male *S. lanzhouensis* were sequenced and assembled at the chromosome level. A male-specific region of the Y chromosome (MSY) on Chr24 was identified, which harbors a potential sex-determining gene, *amhr2y*. Loss-of-function of *amhr2y* in XY individuals resulted in male-to-female sex reversal in *S. lanzhouensis*. Comparison of the sex chromosomes of *S. lanzhouensis* (Chr24), *S. meridionalis* (Chr24), and *S. asotus* (Chr5) identified the sex-determining gene origin, repeat sequence expansion, gene gathering and degeneration in sex-determining region, and sex chromosome turnover in *Silurus*, providing a clear case for the early evolutionary trajectory of homomorphic sex chromosomes.

## Results

### Sequencing and Assembly of XX and YY Genomes

**Fig. 1.**
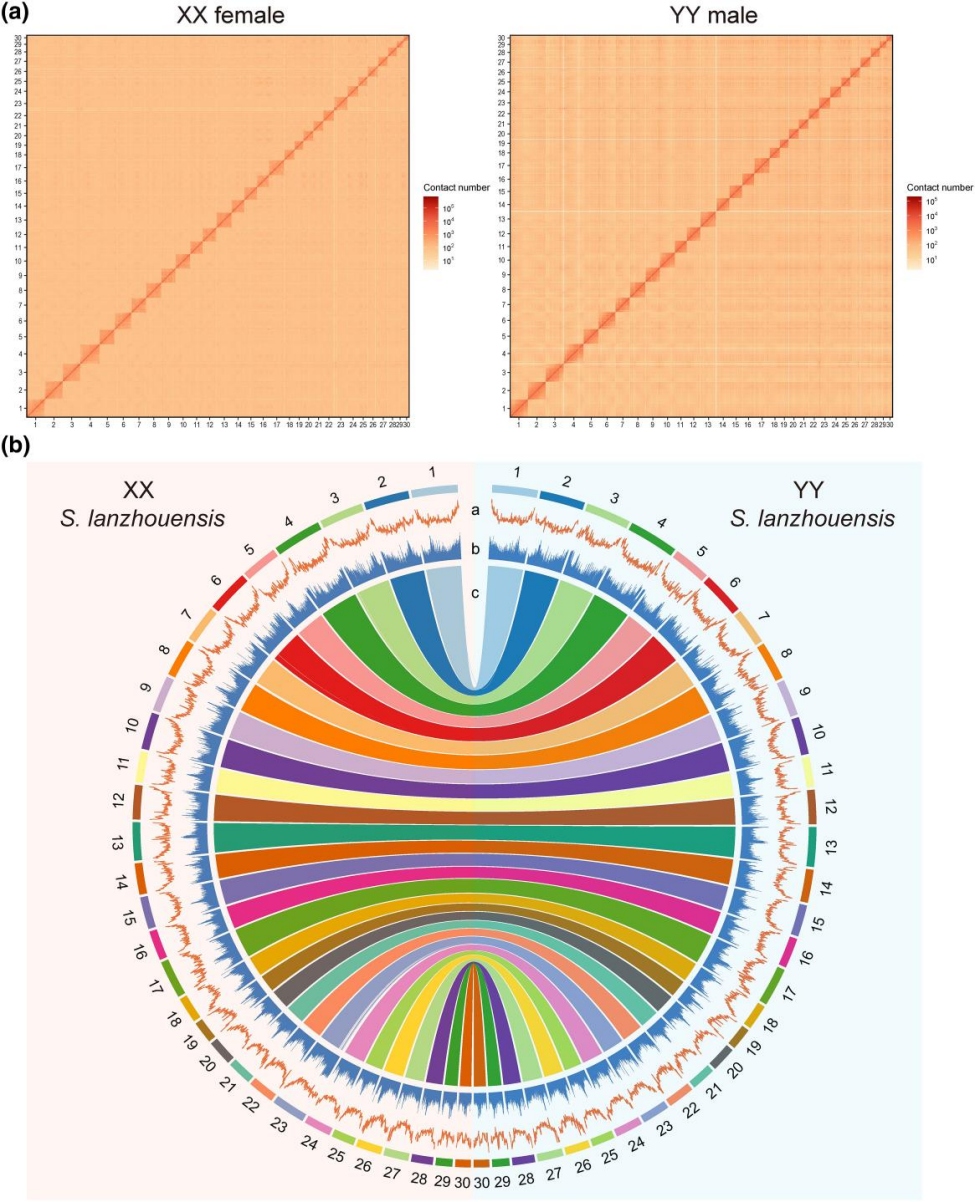
Genome assemblies of an XX female and YY male in *S. lanzhouensis*. a) Chromosomal contact matrix of the XX and YY genome assemblies using high-throughput chromatin conformation capture (Hi-C) data. The density of the Hi-C interactions is indicated by the color bar on the right. b) Genomic comparison between the XX and YY genome assemblies. The Circos plot shows the assembled chromosomes, GC content (a), repeat content (b), and synteny relationship (c).

An XX female from an artificial gynogenetic family and a YY male from a self-fertilized family of *S. lanzhouensis* (Wang et al. 2021), both of which exhibit high genetic homozygosity (Niu et al. 2024), were chosen for genomic sequencing and assembly. The size of the *S. lanzhouensis* genome was estimated to be 771.35 Mb with 0.25% heterozygosity by *k*-mer analysis (supplementary fig. S1, Supplementary Material online). Using PacBio continuous long reads, Illumina short reads, and high-throughput chromatin conformation capture (Hi-C) sequencing technologies (supplementary table S1, Supplementary Material online), 2 high-quality genome assemblies were generated for the XX female and YY male *S. lanzhouensis* (supplementary fig. S2, Supplementary Material online). The haplotype genome of XX female was 774.75 Mb with scaffold N50 of 28.89 Mb, containing 97.0% complete benchmarking universal single-copy ortholog (BUSCO) genes, 24,836 protein-coding genes, and 324.84 Mb repeat contents (supplementary table S2 to S4, Supplementary Material online). The haplotype genome of YY male was 776.88 Mb with scaffold N50 of 29.43 Mb, containing 96.6% complete BUSCO genes, 24,948 protein-coding genes, and 323.09 Mb repeat contents (supplementary table S2 to S4, Supplementary Material online). A total of 99.8% sequences of the XX haplotype genome (773.14 Mb) and 99.9% sequences of the YY haplotype genome (776.18 Mb) were both anchored to 30 chromosomes (Fig. 1; supplementary table S5, Supplementary Material online). And the chromosome number in the 2 genome assemblies is consistent with the previous studies (Wang et al. 2021; Yang et al. 2023). The genome-wide chromosomal contact matrices of both genome assemblies show continuous and strong Hi-C interaction signals within chromosomal groups (Fig. 1a). Extremely high sequence synteny was found between the genomic sequences of XX and YY *S. lanzhouensis* (Fig. 1b).

### Sex Chromosome and Candidate Sex-Determining Gene

**Fig. 2.**
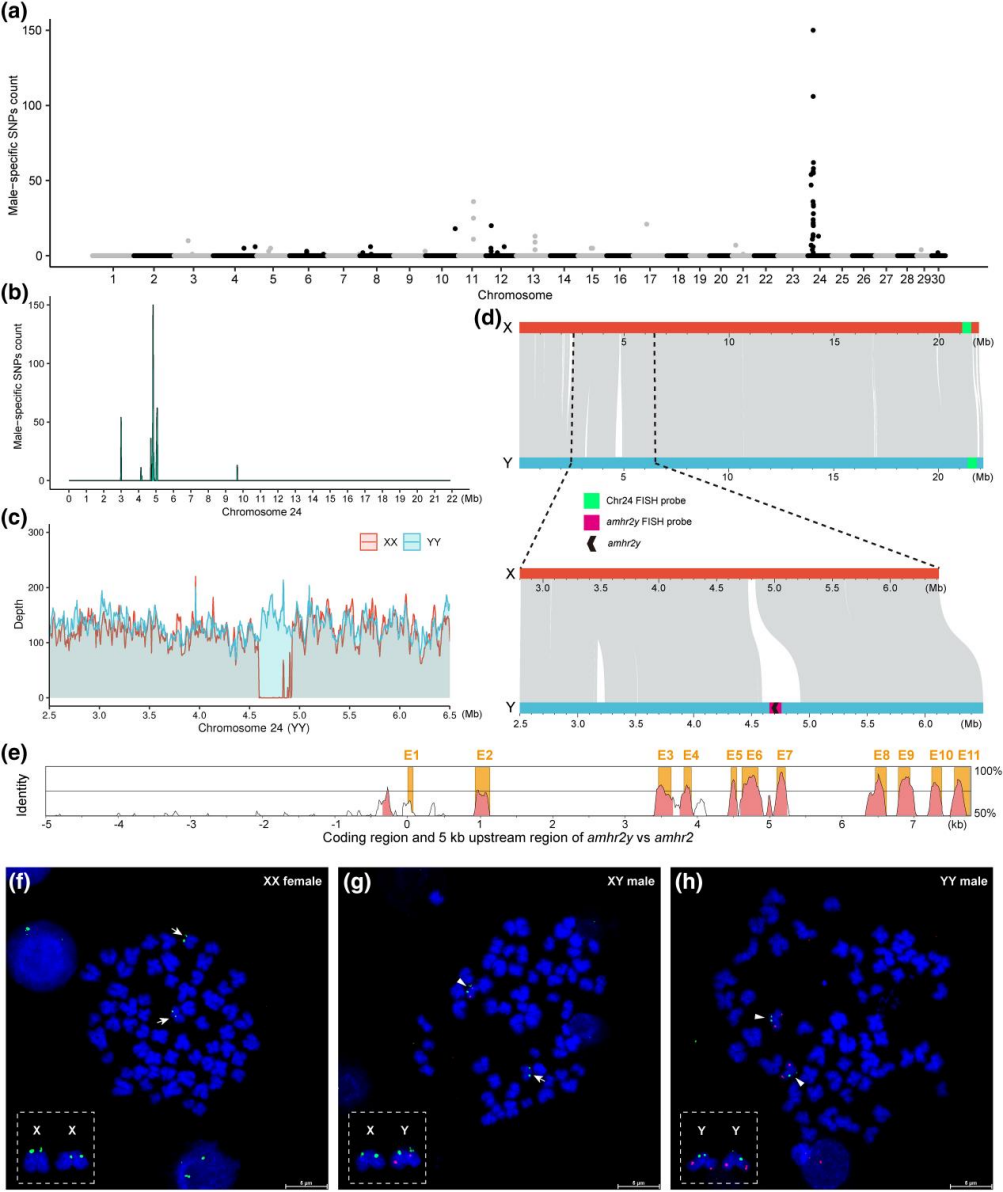
Identification of the sex chromosome and candidate sex-determining gene in *S. lanzhouensis*. a) Distribution of the male-specific SNPs. The distribution of these SNPs was calculated using a 20 kb sliding window with an output point every 10 kb. b) Male-specific SNP distribution on Chr24. c) Coverage depths of PacBio long reads for XX female (red) and YY male (blue) in an approximately 4.0 Mb region enriched with male-specific SNPs on Chr24 of the YY *S. lanzhouensis* assembly. d) Syntenic comparison between X and Y chromosomes. The Chr24 and *amhr2y* probes used in FISH are highlighted in green and purple, respectively. e) Sequence comparison between *amhr2* and *amhr2y*, with *amhr2* as the reference. E1–E11 indicate the 11 exons. f to h) FISH analysis of the sex chromosome and sex-determining gene in the metaphases of XX female (f), XY male (g), and YY male (h). X chromosomes are labeled with the Chr24 FISH probe (green), and Y chromosomes are labeled with the *amhr2y* FISH probe (red) and are indicated by arrows and arrowheads, respectively.

To identify the sex chromosome and sex-determining gene of *S. lanzhouensis*, we analyzed the sex-specific SNP distribution using resequencing data from 18 XX females and 11 XY males (accession number: CRA010634; National Genomics Data Center). A total of 1,071,828 high-quality SNPs were identified using the XX genome as a reference. Of these, 673 (0.063%) were identified as male-specific SNPs (supplementary fig. S3a and table S6, Supplementary Material online). However, no female-specific SNP was identified. It is notable that 478 (71.0%) of the male-specific SNPs were located on Chr24, while the remaining 195 (29.0%) SNPs were distributed across the other 16 chromosomes (Fig. 2a; supplementary fig. S3b, Supplementary Material online). This indicates that Chr24 is the sex chromosome (X or Y) of *S. lanzhouensis*. Moreover, male-specific SNPs on Chr24 were concentrated mainly in the 2.5 to 6.5 Mb region of this chromosome (Fig. 2b; supplementary table S6, Supplementary Material online). Alignments of the PacBio long reads from the XX and YY individuals to the YY genome assembly revealed an approximately 320 kb MSY range from 4.60 to 4.92 Mb region of the Y chromosome (Fig. 2c). The MSY contained an annotated gene *amhr2y* (Fig. 2d), which is a duplicate copy of the *amhr2* gene found on the autosome Chr8 (Fig. 2e). Both *amhr2y* and *amhr2* contain 11 exons and 10 introns (supplementary fig. S4a, Supplementary Material online), and the cDNA sequences and deduced amino acid sequences of *amhr2y* and *amhr2* shared 78.68% and 70.56% identity, respectively (supplementary fig. S4b and c, Supplementary Material online).

To confirm the male specificity of *amhr2y*, we designed primer pairs specific to *amhr2y* and *amhr2* and common to both. The amplification band of *amhr2y* was detected in all the XY and YY males, but not in the XX females (supplementary fig. S4b and d, Supplementary Material online). Fluorescence in situ hybridization (FISH) was performed on XX females, XY males, and YY males of *S. lanzhouensis* using 2 DNA probes: a Chr24-specific DNA sequence shared by the X and Y chromosomes and *amhr2y* and its upstream and downstream sequences (Fig. 2d). The Chr24-specific DNA probe produced signals on both the X and Y chromosomes, whereas the *amhr2y* probe signal was detected only on the Y chromosome (Fig. 2f to h). These data confirm that *S. lanzhouensis* has an XX/XY sex determination system and that *amhr2y* is a male-specific gene. As *amhr2* has frequently been recruited as a sex-determining gene, especially after it was identified as a male-determining gene in *S. meridionalis* (Zheng et al. 2022) and a candidate male-determining gene in *S. asotus* (Zheng et al. 2023), it is likely that *amhr2y* is a candidate sex-determining gene in *S. lanzhouensis*.

### *Amhr2y* Is Necessary for Male Sex Determination

**Fig. 3.**
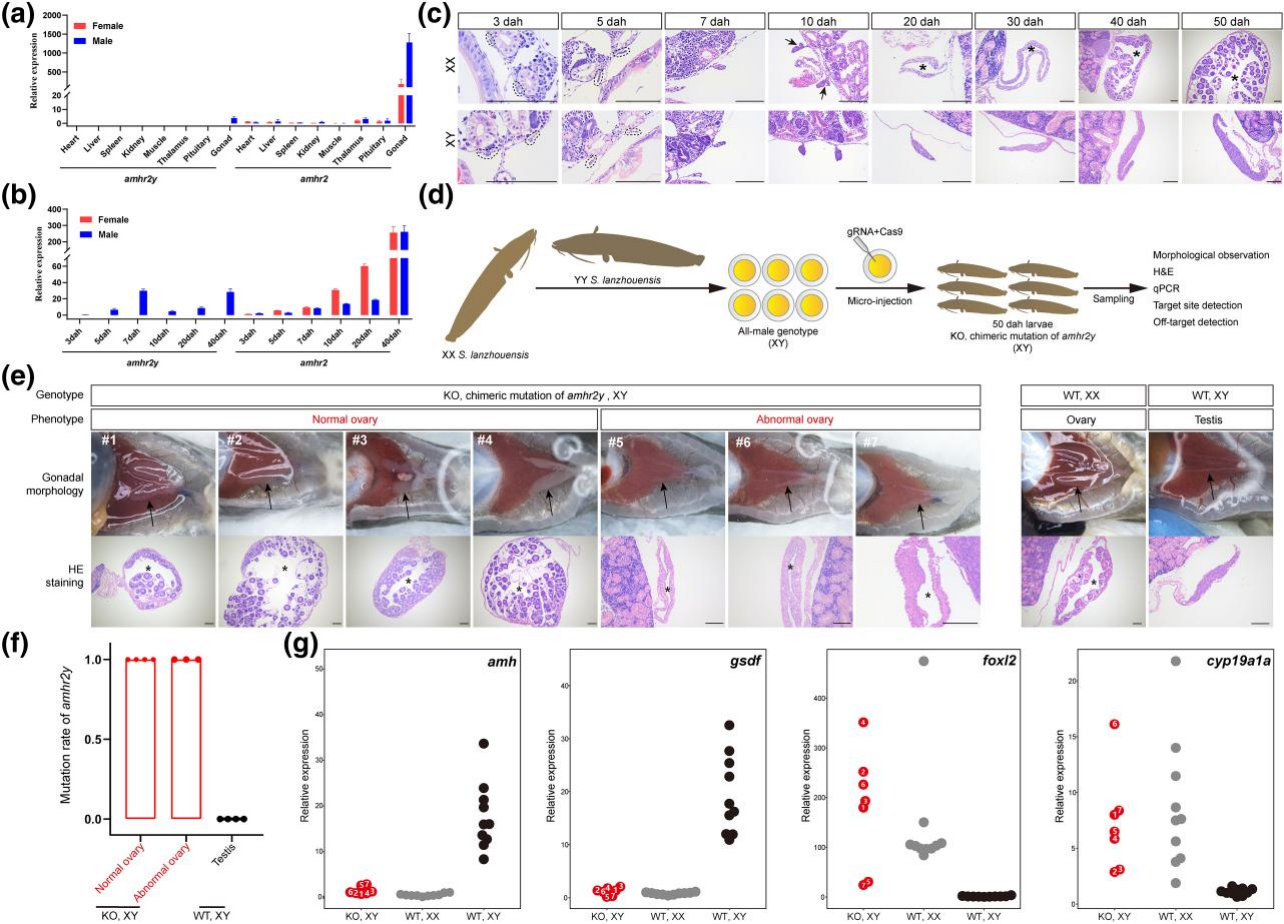
Functional analyses of *amhr2y* in *S. lanzhouensis*. a and b) qPCR of *amhr2y* and *amhr2* in different organs of *S. lanzhouensis* at 6 months old a) and in gonads at different developmental stages b). c) Histological images of gonads at different developmental stages. Black-dotted lines indicate the gonads at 3 and 5 dah; arrows indicate the appearance of tissue outgrowth in female gonads; asterisks indicate ovarian cavities. Scale bars are 100 µm. d) Diagram of the knockout experimental design. e) Gonadal morphology and hematoxylin and eosin staining of gonads in 7 *amhr2y* knockout individuals and WT females and males at 50 dah. Typical normal ovaries with ovarian cavities and developing oocytes (#1, #2, #3, and #4) and abnormal ovaries with empty ovarian cavities (#5, #6, and #7). Arrows indicate the position of the gonads; asterisks indicate ovarian cavities. Scale bars are 100 µm. f) *amhr2y* mutation rates of gonads from knockout and WT groups. g) Transcriptional expression of female and male marker genes, *amh*, *gsdf*, *foxl2*, and *cyp19a1a*, in 7 knockout individuals, 10 WT females, and 10 WT males. Each knockout individual is labeled with the corresponding number from e).

Relative real-time quantitative polymerase chain reaction (qPCR) was used to analyze the expression of *amhr2y* transcripts in 8 organs of *S. lanzhouensis* at 6 months old. At this age, the female and male gonads were fully differentiated and no egg yolk was accumulated in the ovaries. The *amhr2y* transcripts were detected exclusively in male gonads, whereas the *amhr2* transcripts were distributed mainly in both female and male gonads. The expression of *amhr2* was much higher in testes than it was in ovaries (Fig. 3a). During the early developmental stages of gonads, *amhr2y* expression was detected exclusively in male gonads and reached a peak at 7 days after hatching (dah) (Fig. 3b). This is the crucial period for sex determination because gonadal morphological differentiation between females and males occurs at 10 dah (Fig. 3c). Conversely, *amhr2* was expressed in both female and male gonads during the early developmental stages (Fig. 3b).

As *amhr2y* is a Y chromosome–specific gene and may have a male-determining function, we performed a loss-of-function analysis using CRISPR/Cas9 in an all-male family with an XY genotype. The family was derived from a wild-type (WT) XX female and a YY male (Fig. 3d). Two *amhr2y* gRNAs for 2 target sites (supplementary fig. S4a and b, Supplementary Material online) were coinjected with Cas9 protein into fertilized eggs. At 50 dah, we randomly selected 7 genotypic male individuals (XY genotype) and examined their gonadal phenotypes in the G0 population with chimeric *amhr2y* mutations. Of the 7 individuals examined, 4 developed typical normal ovaries with ovarian cavities and developing oocytes, and the other 3 formed abnormal ovaries with empty ovarian cavities (Fig. 3e). Immunofluorescence analysis of the germ cell–specific protein Vasa showed that the abnormal ovaries had lost almost all the germ cells (supplementary fig. S5, Supplementary Material online), whereas all the WT genotypic males developed normal testes. We examined the *amhr2y* genotypes of the 7 individuals by PCR amplification and clone sequencing. A total of 10 clones were sequenced for each individual, and all of the sequenced clones showed deletions and/or insertions in comparison to WT genotype (supplementary fig. S6, Supplementary Material online). The absence of a WT sequence indicates that *amhr2y* was completely mutated in the gonads of the sex-reversed individuals (Fig. 3f). The transcriptional expression levels of key male-related (*amh* and *gsdf*) (Liu et al. 2022; Wang et al. 2022a, 2022b, 2022c) and female-related genes (*foxl2* and *cyp19ala*) (Yang et al. 2017; Wu et al. 2020; Gan et al. 2021) in the gonads of the sex-reversed individuals were similar to those in the gonads of WT females (Fig. 3g). These results demonstrate that *amhr2y* is necessary for male sex determination of *S. lanzhouensis*.

### Sex-Determining Gene Origin and Chromosome Turnover in *Silurus* Species

**Fig. 4.**
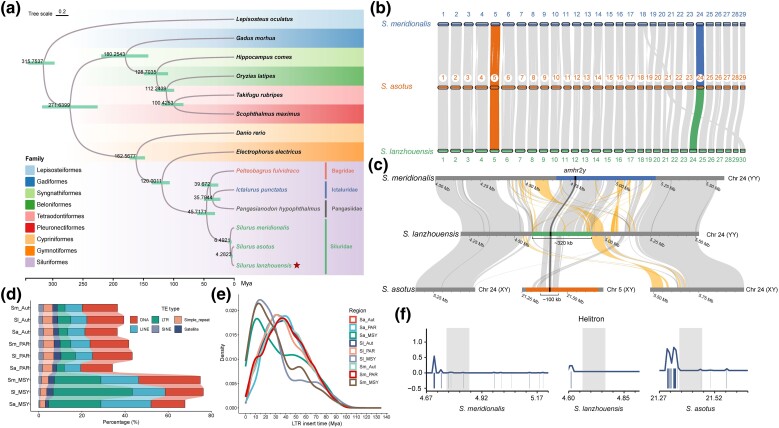
Sex chromosome evolution and expansion of repeats in *Silurus* fishes. a) Phylogenetic relationship of 3 *Silurus* fishes and 11 other teleost fishes. The divergence time was labeled at the nodes, and the green rectangle at each node indicates the 95% confidence interval. b) Genome-scale synteny relationship of *S. lanzhouensis*, *S. meridionalis*, and *S. asotus*. Lines linking 2 chromosomes indicate 1-to-1 correspondence of homologous regions. The homologs of autosomes are linked with gray lines, and the homologs of sex chromosomes are connected within multicolored lines. c) Synteny relationship of the MSY and adjacent PAR of 3 *Silurus* fishes. Synteny relationships of syntenic and reverse regions are shown in gray and yellow, respectively. The MSYs and *amhr2y* gene were highlighted in different color. d) Bar chart showing the proportions of several repeat families in autosomes, PARs, and MSYs of 3 *Silurus* fishes. LTR, long terminal repeat; LINE, long interspersed element; SINE, short interspersed element. e) LTR insertion time analysis in autosomes, PARs, and MSYs of 3 *Silurus* fishes. f) Helitron distribution around the conserved approximately 100 kb block of 3 *Silurus* fishes. Gray column indicates the 100 kb block.

To determine the evolutionary relationship of *Silurus* fishes, we conducted phylogenetic analysis using single-copy orthologs. The phylogenetic tree showed that *S. lanzhouensis* was more closely related to *S. asotus* than it was to *S. meridionalis*. The divergence of *S. meridionalis* from *S. asotus* and *S. lanzhouensis* occurred approximately 6.49 Mya, while the *S. asotus* and *S. lanzhouensis* branches split approximately 4.28 Mya. The 3 *Silurus* catfishes diverged from the other 3 Siluriformes families (Bagridae, Ictaluridae, and Pangasiidae) approximately 45.72 Mya (Fig. 4a). *Silurus lanzhouensis* has 60 chromosomes, whereas *S. meridionalis* and *S. asotus* have 58 chromosomes (Fig. 4b). Recently, Chr24 of *S. meridionalis* (Zheng et al. 2022) and Chr5 of *S. asotus* (Zheng et al. 2023) were identified as sex chromosomes containing the candidate sex-determining gene *amhr2y*. In *S. lanzhouensis*, the sex-determining gene *amhr2y* was also located on Chr24.

Comparison of the MSYs of the 3 *Silurus* species showed that *S. meridionalis* and *S. lanzhouensis* share a homologous block of approximately 320 kb, whereas *S. asotus* only shares a homologous block of approximately 100 kb with *S. meridionalis* and *S. lanzhouensis* (Fig. 4c). Within the 100 kb blocks among the 3 *Silurus* species, the synteny and identity are higher between *S. meridionalis* and *S. lanzhouensis* than those between *S. asotus* and *S. lanzhouensis* (supplementary fig. S7, Supplementary Material online). Subsequently, a synteny analysis of Chr5 was conducted across the 3 species, which revealed that the *amhr2y*-containing insertion was present only on the Y chromosome (Chr5) of *S. asotus*, but absent from the Chr5 of *S. meridionalis* and *S. lanzhouensis* (supplementary fig. S8, Supplementary Material online). These results suggest that the *amhr2* duplicate was initially translocated to Chr24 of the *Silurus* ancestor, resulting in the formation of *amhr2y* and making Chr24 a sex chromosome. In *S. meridionalis* and *S. lanzhouensis*, Chr24 remains the sex chromosome. However, in *S. asotus*, a sex chromosome turnover occurred due to the translocation of an approximately 100 kb block from Chr24 to Chr5, resulting in Chr5 becoming a sex chromosome. Besides, a phylogenetic analysis of *amhr2* and *amhr2y* in catfish was performed to confirm the origin of *amhr2y* in *Silurus* species. The *amhr2y* of the *Silurus* species formed a sister clade to the *amhr2* of the *Silurus* species (supplementary fig. S9, Supplementary Material online). The *Silurus* clade is distinct from the Pangasiidae *amhr2y* clade, indicating that the origin of *amhr2y* in *Silurus* species is independent of *amhr2y* origin in the Pangasiidae family as previously reported (Zheng et al. 2023).

Comparison of the Y and X chromosomes showed that low-identity sequences are mainly surrounding the MSYs, and the remaining regions of the sex chromosomes showed a high degree of identity (supplementary fig. S10, Supplementary Material online), which were defined as the pseudo autosomal regions (PARs). Repeat analysis showed that the long terminal repeats (LTRs) and long interspersed elements (LINEs) were expanded in the MSYs of these 3 *Silurus* species, compared to autosomes and PAR of the Y chromosomes (Fig. 4d). The peak period of LTR insertion of MSYs in *S. meridionalis*, *S. lanzhouensis*, and *S. asotus* was 14.4, 13.0, and 11.3 Mya, respectively (Fig. 4e), indicating that the occurrence of *amhr2y* on Chr24 of the *Silurus* ancestor might be earlier than 14.4 Mya. Furthermore, Helitron repeats were observed to be concentrated around the translocated 100 kb block in the MSY of *S. asotus*, in contrast to their distribution in the MSYs of *S. meridionalis* and *S. lanzhouensis* (Fig. 4f). Helitron transposons, which are widely distributed in all eukaryotes, frequently capture and move DNA fragments (Kapitonov and Jurka 2007). This drives the evolution of the host genome, such as novel gene formation, genetic diversity creation, and sex chromosome turnover (Morgante et al. 2005; Yang et al. 2021). Therefore, the accumulation of Helitron repeats in the MSY might be associated with the sex chromosome turnover of *S. asotus*.

### Gene Gathering and Degeneration in the MSY

**Fig. 5.**
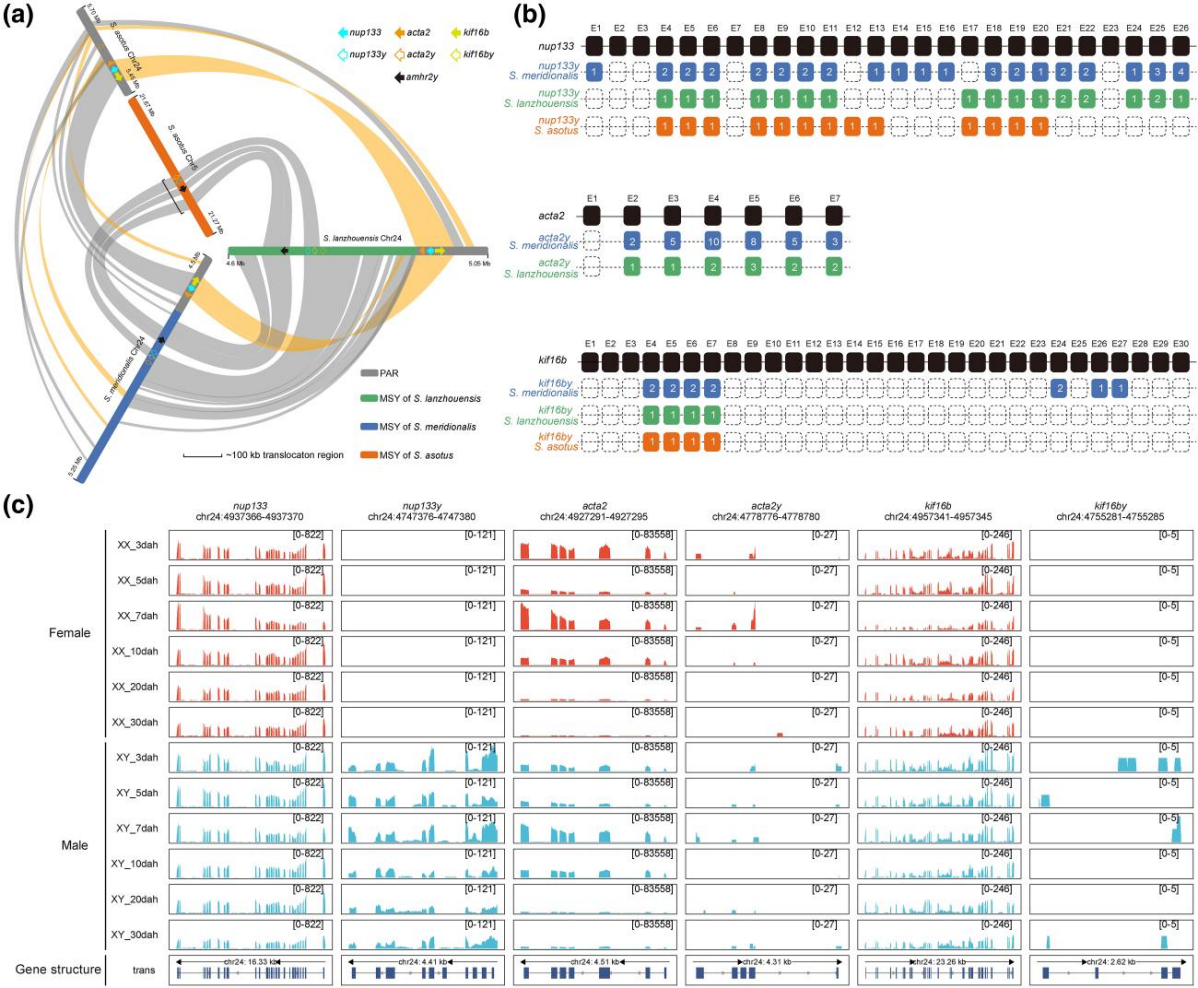
Gene accumulation and degeneration in the MSYs of *Silurus* fishes. a) Chromosomal locations of accumulated genes and their ancestral genes in 3 *Silurus* fishes. The solid arrows represent ancestral genes (*nup133*, *acta2*, and *kif16b*), and the hollow arrows represent accumulated duplicates in Y-specific regions (*nup133y*, *acta2y*, and *kif16by*). b) Schematic diagram of gene structure of *nup133y*, *acta2y*, and *kif16by* and their ancestral genes. The solid and hollow boxes represent present exons and lost exons, respectively. The number in the solid box represents the copy number of this exon. c) RNA-seq coverage of *nup133y*, *acta2y*, and *kif16by* and their ancestral genes in gonadal samples of *S. lanzhouensis* at 3, 5, 7, 10, 20, and 30 dah.

Except the sex-determining gene *amhr2y*, we also identified other gene fragments in the MSYs of *Silurus* species. In the MSYs of *S. meridionalis* and *S. lanzhouensis*, we detected fragmental duplicates of *nup133* (nucleoporin 133), *acta2* (actin alpha 2), and *kif16b* (kinesin family member 16b), which were named as *nup133y*, *acta2y*, and *kif16by*, respectively. However, in the MSY of *S. asotus*, only *nup133y* and *kif16by* were detected near *amhr2y* (Fig. 5a). *Acta2y* was located outside the approximately 100 kb block that mediates sex chromosome turnover, so the translocation of the 100 kb block from the Chr24 to Chr5 resulted in the loss of *acta2y* in the MSY of Chr5 in *S. asotus* (Fig. 5a).

*Nup133*, *acta2*, and *kif16b* are neighboring genes located in the nonsex-specific region of Chr24 and contain 26, 7, and 30 exons, respectively. *Nup133y*, *acta2y*, and *kif16by* were derived from *nup133*, *acta2*, and *kif16b*, respectively, by duplication and translocation in the common ancestor of *S. meridionalis*, *S. lanzhouensis*, and *S. asotus*. The MSY of *S. meridionalis* exhibited amplification of some exons of *nup133y*, *acta2y*, and *kif16by*. Also, some amplified exons of *nup133y* and *acta2y* were identified in the MSY of *S. lanzhouensis*. The exons of *nup133y*, *acta2y*, and *kif16by* display insertions, deletions, and synonymous and nonsynonymous mutations when compared to the corresponding exons of their ancestral genes (supplementary tables S7 to S14, Supplementary Material online). Meanwhile, the *nup133y* and *kif16by* genes of *S. meridionalis*, *S. lanzhouensis*, and *S. asotus*, as well as the *acta2y* gene of *S. meridionalis* and *S. lanzhouensis*, have experienced a process of genetic degeneration, resulting in the loss of certain exons and the absence of an intact genome structure (Fig. 5b).

The expression patterns of *nup133*, *acta2*, and *kif16b* transcripts were similar in female and male *S. lanzhouensis* during early gonadal developmental stages (Fig. 5c). Unlike *nup133*, *nup133y* showed male-specific transcriptional expression, but its expression level was significantly lower than that of *nup133*. The *acta2y* and *kif16by* transcription was barely detected in early gonads (Fig. 5c). The low expression or nonexpression of the MSY genes may be due to gene degeneration along with sex chromosome evolution.

## Discussion

Fish species with newly formed sex chromosomes provide an ideal system to trace the evolutionary trajectory of sex chromosomes at early stages, unlike Y and W chromosomes in most studied mammals and birds that have long origin histories and high degeneration. In most fish, sex chromosomes are young and homomorphic, making it challenging to accurately assemble X and Y chromosomes without chimeric sequence from an XY individual. In this study, we used an XX female from an artificially gynogenetic family and a YY male from a self-fertilized family (Wang et al. 2021) for whole-genome sequencing in *S. lanzhouensis*. Artificial gynogenesis and self-fertilization both increased the genetic homozygosity of the XX female and YY male (Niu et al. 2024), making them ideal materials for assembling pure X and Y chromosomes and for investigating the early evolution of sex chromosomes. In addition, the genome assemblies of XX and YY individuals also provide valuable genomic resources for the genetic breeding of *S. lanzhouensis*.

**Fig. 6.**
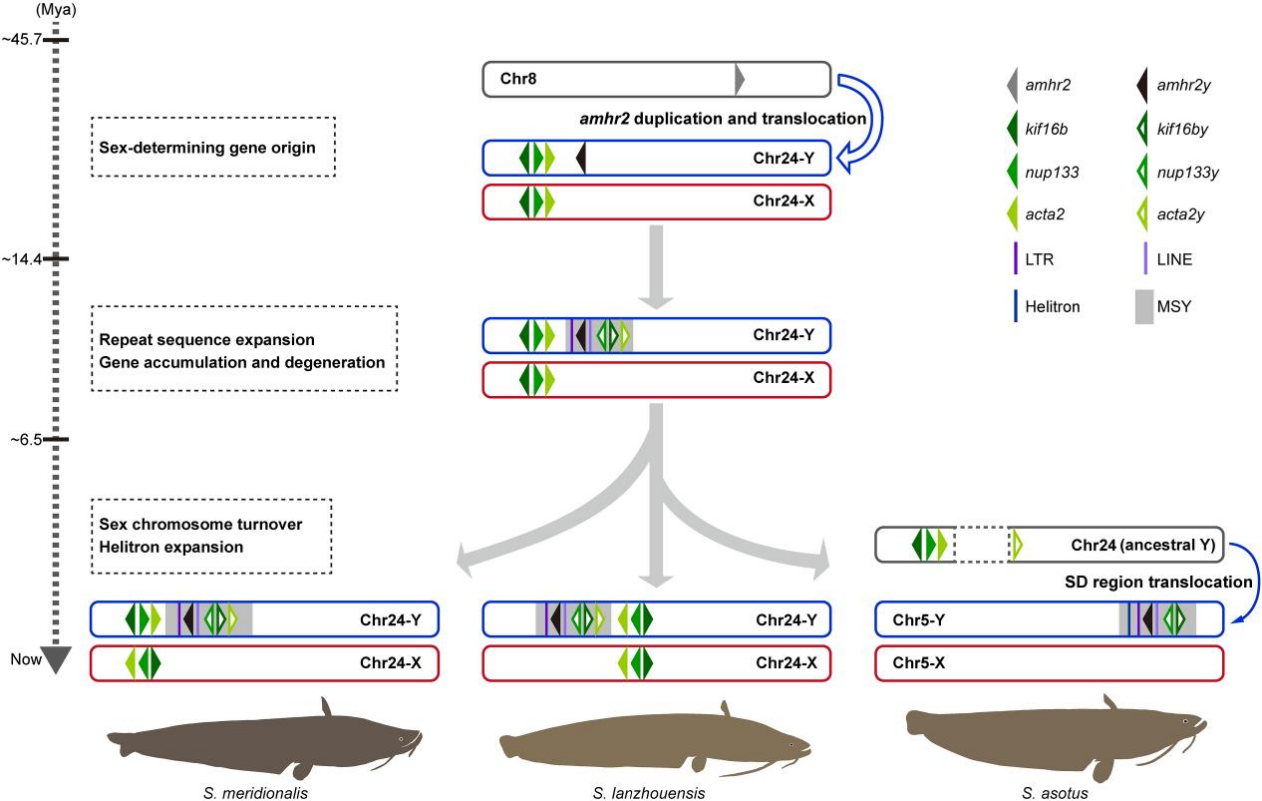
Schematic diagram of sex chromosome evolution in *Silurus* fish. MSY, male-specific region of the Y chromosome; SD region, sex-determining region; Chr, chromosome.

Sex chromosomes exhibit remarkable diversity due to independent origins and frequent turnovers. Diverse candidate sex-determining genes have been identified in catfish (Siluriformes), including *pfpdz1* (PDZ domain–containing gene) of yellow catfish (*Pelteobagrus fulvidraco*) (Dan et al. 2018), *bcar1* (breast cancer anti-resistance 1) of channel catfish (*Ictalurus punctatus*) (Bao et al. 2019), and *amhr2y* in the Pangasiidae family (Wen et al. 2022). The phylogenetic relationship suggested that the *amhr2y* gene is a conserved candidate master sex determinant, and these orthologs of *amhr2y* share a common ancestral origin in the Pangasiidae family (Wen et al. 2022). Compared to the *amhr2y* gene on Chr7 of Pangasiidae family, *amhr2y* is located on different chromosomes in *Silurus* species (Chr24 of *S. lanzhouensis/S. meridionalis* and Chr5 of *S. asotus*). Besides, *amhr2y* of *Silurus* species clustered as a sister clade to *amhr2* of *Silurus* species (supplementary fig. S9, Supplementary Material online), and a conserved block was observed among the MSYs of *Silurus* species, but no collinearity was detected in the MSYs of *Silurus* and Pangasiidae catfish (Zheng et al. 2022, 2023). These results indicate that the recruitment of *amhr2y* as a male-determining gene in *Silurus* species is independent of that in the Pangasiidae family (Zheng et al. 2023). However, the evolutionary process that led to the location of *amhr2y* on different sex chromosomes in *Silurus* species remains unclear. This study identifies the sex chromosome and sex-determining gene in *S. lanzhouensis* and reveals the dynamic evolutionary trajectory of sex chromosomes in *Silurus* catfish (Fig. 6).

In the common ancestor of *S. meridionalis*, *S. lanzhouensis*, and *S. asotus*, the *amhr2* gene was duplicated and translocated from Chr8 to Chr24. This event was accompanied by an expansion of LTRs and LINEs and the accumulation of duplicates of *nup133*, *acta2*, and *kif16b* near *amhr2y*. Subsequently, due to repeat insertion in MSY, *nup133y*, *acta2y*, and *kif16by* degenerated, leading to their fragmentation and reduced expression. In *S. meridionalis* and *S. lanzhouensis*, Chr24 was maintained as sex chromosome, whereas in *S. asotus*, sex chromosome turnover occurred resulting in the transition of sex chromosomes from Chr24 to Chr5. The sex chromosome turnover in *S. asotus* was associated with the amplification of Helitron repeats in MSY. These findings provide a clear understanding of the early evolution of sex chromosomes and sex chromosome turnover (Fig. 6).

Most sex-determining genes are derived from members of the TGF-β signaling pathway by gene duplication or allelic diversification (Pan et al. 2021b; Li et al. 2022), including *amh* (Li et al. 2015), *amhr2* (Qu et al. 2021), *gsdf* (Kabir et al. 2022), *gdf6* (Imarazene et al. 2021), and *bmpr1bb* (Rafati et al. 2020). During key periods of sex determination, all these sex-determining genes derived from members of the TGF-β pathway showed male-specific expression. However, the mechanism underlying sex determination remains unclear. In Atlantic herring (*Clupea harengus*), Bmpr1bby phosphorylates Smads in the absence of ligand Amh and type II receptor Amhr2, suggesting that Bmpr1bby has a different downstream pathway to mediate male determination compared with that of Bmpr1bb (Rafati et al. 2020). Intriguingly, Bmpr1bby in Atlantic herring (Rafati et al. 2020), Amhr2by in the Pangasiidae catfish family (Wen et al. 2022), and Amhr2by in the Percidae family (Kuhl et al. 2023) all have truncated N-terminal domains which lack the N-terminal ligand binding extracellular part. The lack of this N-terminal region may be associated with the evolution of male determination. Further in-depth studies are needed to clarify the regulatory and evolutionary mechanisms of sex-determining genes derived from members of the TGF-β pathway.

In conclusion, we precisely assembled the genomes of homozygous XX female and YY male *S. lanzhouensis* at the chromosome level and identified a sex-determining gene, *amhr2y*, on Chr24. We have showed the early evolutionary trajectory of sex chromosomes in *Silurus* catfish, including sex-determining gene origin, repeat sequence expansion, gene gathering and degeneration in sex-determining region, and sex chromosome turnover. Our findings cover almost all typical events associated with sex chromosome evolution, facilitating a deeper understanding of the diversity and variability of sex determination mechanisms.

## Materials and Methods

### Experimental Animals

The *S. lanzhouensis* used in this study were provided by the National Aquatic Biological Resource Center (NABRC), Institute of Hydrobiology, Chinese Academy of Sciences, Wuhan, China. All the animal experiments were performed in accordance with the principles of the Animal Care and Use Committee of the Institute of Hydrobiology, the Chinese Academy of Sciences.

### Whole-Genome Sequencing

Genomic DNA was extracted from blood of an XX female *S. lanzhouensis* of an artificial gynogenetic family and a YY male *S. lanzhouensis* of a self-fertilized family (Wang et al. 2021). Two continuous long-read libraries were constructed using SMRT bell Template Prep Kits (Pacific Biosciences, USA) for PacBio SMRT sequencing. The libraries were sequenced on a PacBio Sequel II platform with an approximately 20 kb long-read layout. Two paired-end genomic libraries were constructed using a TruSeq Nano DNA LT Sample Preparation Kit (Illumina, USA) for Illumina short-read sequencing. The libraries were sequenced on an Illumina HiSeq X-Ten platform with approximately 150 bp paired-end reads generated. Fresh liver tissue and peripheral blood were collected for Hi-C library preparation using a NEBNext Ultra II DNA library Prep Kit (NEB, USA). The Hi-C libraries were sequenced on an Illumina HiSeq X-Ten platform as described previously (Gong et al. 2022). Library construction and genome sequencing were performed by OE Biotech Co., Ltd. (Shanghai, China).

### Genome Assembly and Quality Evaluation

Before genome assembly of *S. lanzhouensis*, a genome survey was performed as described previously (Wang et al. 2022a, 2022b, 2022c). The following software was separately employed for the primary assembly of XX individual with default parameters, including Falcon (version 1.3.0) (Chin et al. 2016), Canu (version 2.2) (https://github.com/marbl/canu), wtdbg2 (version 2.5) (https://github.com/ruanjue/wtdbg2), Flye (version 2.9.2) (https://github.com/fenderglass/Flye), and MECAT2 (version 20190314) (https://github.com/xiaochuanle/MECAT2). Purge_dups (version 1.2.6) (https://github.com/dfguan/purge_dups) was used to remove haplotigs and contig overlaps. Based on the consistency between the assembly size and the genome survey size (771.35 Mb), as well as the number of contigs (supplementary table S15, Supplementary Material online), Falcon was selected as the optimal assembler for the XX and YY fish assemblies, with subsequent polishing and scaffolding. The PacBio long reads were mapped back to the Falcon assembly using Minimap2 (version 2.18) (Li, 2018) and polished using Arrow (version 2.0.0). Subsequently, the Illumina short reads were used in 2 rounds of consensus correction by Pilon (version 1.23) (Walker et al. 2014). The clean Hi-C reads were aligned to the polished Falcon assembly, and raw inter/intrachromosomal contact maps were built using BWA-MEM (version 0.7.17) (Li 2013) with default parameters. Then, the Juicer pipeline (version 1.5.7) (Durand et al. 2016) and 3D-DNA pipeline (version 180922) (Dudchenko et al. 2017) were used with default parameters to cluster, orient, and correct all the contigs. The resulting assembly was visualized using Juicebox (version 1.11.9) (Dudchenko et al. 2018).

We assessed the quality of the final assembly using various measures. To assess continuity, contig and scaffold N50 were calculated using assembly-stats (version 1.0.1) (https://github.com/sanger-pathogens/assembly-stats). The *k*-mer-based consensus quality value and completeness were measured using Merqury (version 1.3) (Rhie et al. 2020) with 19-mers. BUSCO (version 5.4.7) (Manni et al. 2021) was used to assess the completeness of the genome assembly with the “actinopterygii_odb10” data set.

### Genome Annotation

Repeats and protein-coding genes of *S. lanzhouensis* were annotated using methods described previously (Gong et al. 2018). For repetitive element predictions, Tandem Repeats Finder (version 4.09) (Benson 1999) was used to identify tandem repeat elements, and then, RepeatModeler (version 2.04) (https://www.repeatmasker.org/RepeatModeler/) was used to build a de novo repeat library of *S. lanzhouensis*. Finally, transposable elements were identified and located using RepeatMasker (version 4.15) (https://www.repeatmasker.org/RepeatMasker/) based on homology searches against the Repbase library (Bao et al. 2015) and de novo library.

Protein-coding genes were annotated using de novo, homology-based, and transcriptome-based prediction software. For de novo annotation, Augustus (version 3.4.0) (Stanke et al. 2006) and Helixer (version 0.3.2) (Stiehler et al. 2020) were employed to predict the protein-coding gene structures. For homology-based annotation, protein sequences of the closely related species *S. meridionalis* (GCA_014805685.1) were downloaded from the National Center for Biotechnology Information (NCBI) and aligned to the *S. lanzhouensis* genome assembly using Miniprot (version 0.11) (Li 2023). For transcriptome-based annotation, the transcriptome data were mapped onto the genome assembly using HISAT2 (version 2.21) (Kim et al. 2019), and then, the transcripts were assembled by StringTie2 (version 2.21) (Kovaka et al. 2019). Finally, EVidenceModeler (version 2.1.0) (Haas et al. 2008) was used to integrate the 3 gene prediction and annotation results.

### Identification of Sex Chromosome and Y-Specific Region

The whole-genome resequencing data of 18 XX females and 11 XY males of *S. lanzhouensis* (accession number: CRA010634, National Genomics Data Center) (Niu et al. 2024) were used to detect sex-specific SNPs using a method described previously (Gong et al. 2022). The 18 XX females used for resequencing were sampled from 3 families (Niu et al. 2024). The first family is an artificial gynogenetic family, derived from an XX female. The second family was a self-fertilized family, derived from an XY hermaphrodite individual. The third family was a sexually reproduced family, derived from an XX female and an XY male. The 11 XY males used for resequencing were derived from the same self-fertilized family and sexually reproduced family as the XX females. We aligned resequencing data from 18 XX females and 11 XY males to our XX reference genome using BWA-MEM. The variants were called using the GATK pipeline (version 4.2.5.0) (McKenna et al. 2010). For male-specific SNP identification, we employed a custom script (https://github.com/GRGong/SexSNPFinder) to extract SNPs that were homozygous reference (0/0) in all females (18 out of 18 males) and heterozygous (0/1) in all males (11 out of 11 males) when mapped to the XX genome. For female-specific SNP identification, we looked for sites that were heterozygous (0/1) in all females (18 out of 18 males) and either homozygous reference (0/0) or homozygous alternate (1/1) in all males (11 out of 11 males).

To identify the sex chromosome and delimitate the region enriched in male-specific SNPs, we used a 20 kb sliding window with a step of 5 kb to count the number of male-specific SNPs. We performed coverage analysis of long reads to identify the Y-specific region. First, both XX and YY PacBio continuous long reads were remapped to the YY genome assembly using Winnowmap2 (version 2.03) (Jain et al. 2022). Then, Mosdepth (version 0.3.3) (Pedersen and Quinlan 2018) was used to calculate the depth of sequence coverage along the whole assembly. Finally, a 20 kb sliding window with a step of 5 kb was used to compute the average coverage depth and delimitated the Y-specific region based on the coverage difference of the XX and YY reads.

### FISH

The metaphase chromosomes of XX, XY, and YY *S. lanzhouensis* individuals were prepared by kidney cell–phytohemagglutinin (PHA) culture in vivo as previously reported (Lu et al. 2021). The fragments specific to Chr24 (both X and Y chromosomes) and the fragments containing the *amhr2y* gene were amplified by PCR for FISH probes (Fig. 2). The PCR primers are listed in supplementary table S16, Supplementary Material online. FISH analysis was performed as described previously (Li et al. 2016) with minor manual adjustments. The Chr24 and *amhr2y* probes were labeled by digoxigenin (DIG)–Nick Translation Mix (Roche) and biotin–Nick Translation Mix (Roche), respectively. The probes labeled with digoxigenin and biotin were stained with DyLight 488 Anti-Digoxigenin/Digoxin antibody (Vector Labs) and ExtraAvidin-Cy3 antibody (Sigma), respectively. After chromosome staining with DAPI, the images were photographed using a TCS SP8 STED microscope (Leica).

### Sequence Analysis and Transcriptional Expression of *amhr2* and *amhr2y*

Full-length cDNA sequences of *amhr2* and *amhr2y* were obtained by 5′ and 3′ rapid amplification of cDNA ends (RACE) (SMARTer RACE 5′/3′ Kit, Clontech) using a testicular cDNA library. Open reading frames (ORFs) and deduced protein sequences were predicted via ORF Finder (https://www.ncbi.nlm.nih.gov/orffinder/). The signal peptide, transmembrane region, and intracellular Ser/Thr kinase domain were predicted by SMART (Schultz et al. 2000). Protein and cDNA sequences were aligned using Clustal X (version 1.8) (Larkin et al. 2007). Global pairwise alignment of genomic sequences was performed and visualized using the mVISTA LAGAN program (Brudno et al. 2003).

Eight organs, heart, liver, spleen, kidney, muscle, thalamus, pituitary, and gonad, were sampled from female and male *S. lanzhouensis* at 6 months old. In addition, pooled gonads of 30, 25, 20, 15, 13, and 10 individuals were collected at early gonadal developmental stages of 3, 5, 7, 10, 20, and 40 dah, respectively. Fresh samples were collected in RNAprotect Tissue Reagent (Qiagen) or were collected and quickly frozen in liquid nitrogen and then stored at −80 °C until RNA extraction. RNA extraction, cDNA synthesis, and qPCR were performed as previously described (Niu et al. 2024).

### Histological Analysis and Immunofluorescence

The gonad samples were fixed in 4% paraformaldehyde at 4 °C; for 24 h. The fixed samples were dehydrated and embedded in paraffin and then cut into 4 µm thick sections. Hematoxylin and eosin staining and immunofluorescence of Vasa were performed as described previously (Wang et al. 2022a, 2022b, 2022c). Images were captured using an Axio Imager M2 upright fluorescence microscope (Carl Zeiss).

### CRISPR/Cas9-Based Knockout of *amhr2y*

CRISPR/Cas9-based knockout was performed as described previously (Yu et al. 2022). The synthetic gRNAs (400 ng/μL) (supplementary fig. S4, Supplementary Material online) and TrueCut Cas9 Protein v2 (Invitrogen) (400 ng/μL) were coinjected into fertilized eggs derived from an XX female and a YY male. The gynogenetic offspring from the same XX female were used as the female control group, and the untreated offspring from the XX female and the YY male were used as the male control group.

### Phylogenomic Analysis

Whole-genome protein data sets of 6 Siluriformes species and 8 other teleost fishes were obtained from GenBank for the phylogenomic analysis, including *S. lanzhouensis, S. meridionalis* (GCA_014805685.1), *S. asotus* (GCA_024362625.1), *P. fulvidraco* (GCA_022655615.1), *I. punctatus* (GCA_001660625.1), *Pangasianodon hypophthalmus* (GCA_027358585.1), *Lepisosteus oculatus* (GCA_000242695.1), *Gadus morhua* (GCA_902167405.1), *Hippocampus comes* (GCA_001891065.2), *Oryzias latipes* (GCA_002234675.1), *Takifugu rubripes* (GCA_901000725.2), *Scophthalmus maximus* (GCA_022379125.1), *Danio rerio* (GCA_000002035.4), and *Electrophorus electricus* (GCA_013358815.1). OrthoFinder (version 2.5.5) (Emms and Kelly 2015) was used for orthology inference based on protein sequences, and single-copy ortholog gene families shared in the 14 fish species were identified. The phylogeny was inferred using all sites from the concatenated codon-based alignment of orthologous coding sequences. This approach was selected to maximize the phylogenetic signal from both synonymous and nonsynonymous sites, thereby providing a comprehensive evolutionary perspective. Although the use of only synonymous sites can reduce the influence of selection, the incorporation of nonsynonymous sites enables a more robust inference of deep divergences and facilitates the acquisition of crucial evolutionary information. Briefly, the protein sequences of obtained single-copy orthologous groups were aligned by MAFFT (version 7.490) (Katoh and Standley 2013) with parameters –localpair –maxiterate 1,000, and then, the resulting protein alignments were converted to corresponding codon-based nucleotide alignments using PAL2NAL (version 14) (Suyama et al. 2006). Next, the multiple alignment was trimmed using Gblock (version 0.91b) (Talavera and Castresana 2007). To determine the best-fit substitution model, we used ModelFinder (Kalyaanamoorthy et al. 2017) as implemented in IQ-TREE. ModelFinder selected GTR + F + R5 as the optimal model based on the Bayesian information criterion. IQ-TREE (version 1.6.12) (Nguyen et al. 2014) was employed to construct a maximum likelihood tree for the concatenated superalignment with the GTR + F + R5 model and 1,000 bootstrap replicates, using the following options: -m GTR + F + R5 -bb 1,000 -nt 64. Finally, MCMCtree program in the PAML package (version 4.9j) (Yang 2007) was used to determine the divergence times of the 14 fish species. The divergence time calibration was based on 3 fossil calibration points (108.1 to 148.2 Mya between Siluriformes and Gymnotiformes, 132.0 to 170.0 Mya between Siluriformes and Cypriniformes, and 100.0 to 130.0 Mya between Tetraodontiformes and Beloniformes) from TIMETREE (https://timetree.org/).

### Comparative Genome Analyses

Whole-genome alignment between the XX and YY *S. lanzhouensis* genome assemblies was performed using LAST (version 1452) (Kiełbasa et al. 2011). The X chromosome sequences were aligned to the Y chromosome sequences using Winnowmap2 with parameters -c -x asm20 –eqx. The genomes of 3 *Silurus* catfishes (*S. lanzhouensis*, *S. meridionalis*, and *S. asotus*) were employed to perform chromosome collinear analysis using JCVI (MCscan Python version) (Tang et al. 2008). The genome sequences of the 3 *Silurus* catfishes within the MSY and adjacent PAR were aligned using Progressive Cactus (version 2.7.1) (Armstrong et al. 2020), as it is more suitable for aligning sequences from different species. Subsequently, halSynteny (version 2.2) (Krasheninnikova et al. 2020) was used to identify synteny blocks among the aligned genomes, with a minimum block size of 1,000 bp. Further, to obtain a higher resolution view of synteny and to check the identity between the 3 MSY regions, we then employed wfmash (version 0.15.0) (https://github.com/waveygang/wfmash) with parameters -p 50 -n 1 −4 to perform pairwise alignments. To measure sequence identity, the following procedure was employed: the makewindows function of BEDTools (version 2.31.0) (Quinlan and Hall 2010) was used to create 10 kb windows on the reference genome. Subsequently, the liftover function of the rustybam tool (version 0.1.31) (https://github.com/mrvollger/rustybam) was employed to map intervals, while the stats function of rustybam was used to generate statistical data from the alignment. Sequence identity was calculated by dividing the number of matches by the sum of matches and mismatches.

### Repeat Analysis and Estimation of LTR Retrotransposon Insertion Time

RepeatModeler was used to build a universal repeat library for *S*. *meridionalis*, *S*. *lanzhouensis*, and *S*. *asotus* with parameter “-LTRStruct”. Then, RepeatMasker was employed to identify repeat elements separately for each species. To estimate LTR retrotransposon insertion time, divergence values between LTR sequences were corrected using the Jukes-Cantor formula (Jukes and Cantor 1969). Then, insertion age was estimated as *T* = *D*/2*u* (Kimura 1980), where *T* is the insertion time, *D* is the corrected divergence, and *u* is the nucleotide substitution rate. The genome-wide nucleotide divergence between *S*. *lanzhouensis* and *S*. *meridionalis* was calculated using nucmer and dnadiff from the MUMmer package (version 4.0.0) (Marçais et al. 2018) to estimate the substitution rate as described previously (Luo et al. 2023). The resulting substitution rate was determined to be 2.47 × 10^−9^.

### Phylogenetic Analysis of *amhr2* and *amhr2y*

A phylogenetic analysis was performed on the full-length coding sequences of *amhr2* and *amhr2y* in 16 teleosts, comprising 3 *Silurus* species, 12 other Siluriformes species, and *O. mykiss* (outgroup). The *amhr2*/*amhr2y* coding sequences were predicted based on their genomic and protein sequence annotation or retrieved from GenBank (supplementary table S17, Supplementary Material online). Bayesian inference phylogeny was constructed using MrBayes (version 3.2.7a) (Ronquist et al. 2012) under the K2P + G4 model, in which trees and parameters were sampled every 1,000 generations over a total of 1,000,000 generations, the initial 25% of sampled data were discarded as burn-in. The average standard deviation of split frequencies is 0.004816. Multiple sequence alignment, alignment optimization, and best evolutionary model selection were separately executed by MAFFT, Gblocks, and ModelFinder module of PhyloSuite software (version 1.2.3) (Zhang et al. 2020).

### Analysis of Exon Feature of *nup133y*, *acta2y*, and *kif16by*

To analyze the genetic structure of *nup133y*, *acta2y*, and *kif16by* in MSYs of 3 *Silurus* catfishes, each exon sequence of *nup133*, *acta2*, and *kif16b* was aligned against the MSY genome sequences by Blast program in TBtools software (version 2.096) (Chen et al. 2023). The possible exon segment positions were inferred from Blast hits with an expected e-value of less than 1e−5, and hits with lengths less than 25 nucleotides were ignored. Insertion, deletion, and point mutation of *nup133y*, *acta2y*, and *kif16by* were counted from the resulting alignments.

## Supplementary Material

msae169_Supplementary_Data

## Data Availability

The final XX and YY genome assemblies of *S. lanzhouensis* were submitted to Genome Warehouse (GWH) database of the National Genomics Data Center (NGDC, https://ngdc.cncb.ac.cn) under BioProject PRJCA016180 with accession numbers GWHCBHH00000000.1 and GWHERQF00000000, respectively. All the raw sequencing data (including PacBio CLR data, genome survey data, Hi-C data, and RNA-seq data) used in this study had been deposited in the NGDC Sequence Read Archive (SRA) database with accession number CRA015549.
