# Supplementary material for: Genomic Anatomy of Homozygous XX Females and YY Males Reveals Early Evolutionary Trajectory of Sex-determining Gene and Sex Chromosomes in Silurus Fishes
Source: Mol Biol Evol. 2024 Aug 13;41(8):msae169. doi: 10.1093/molbev/msae169 (PMC11350385; doi:10.1093/molbev/msae169)
Supplement: msae169_Supplementary_Data [file msae169_supplementary_data.zip › Supplementary Materials.pdf]

Supplementary Materials

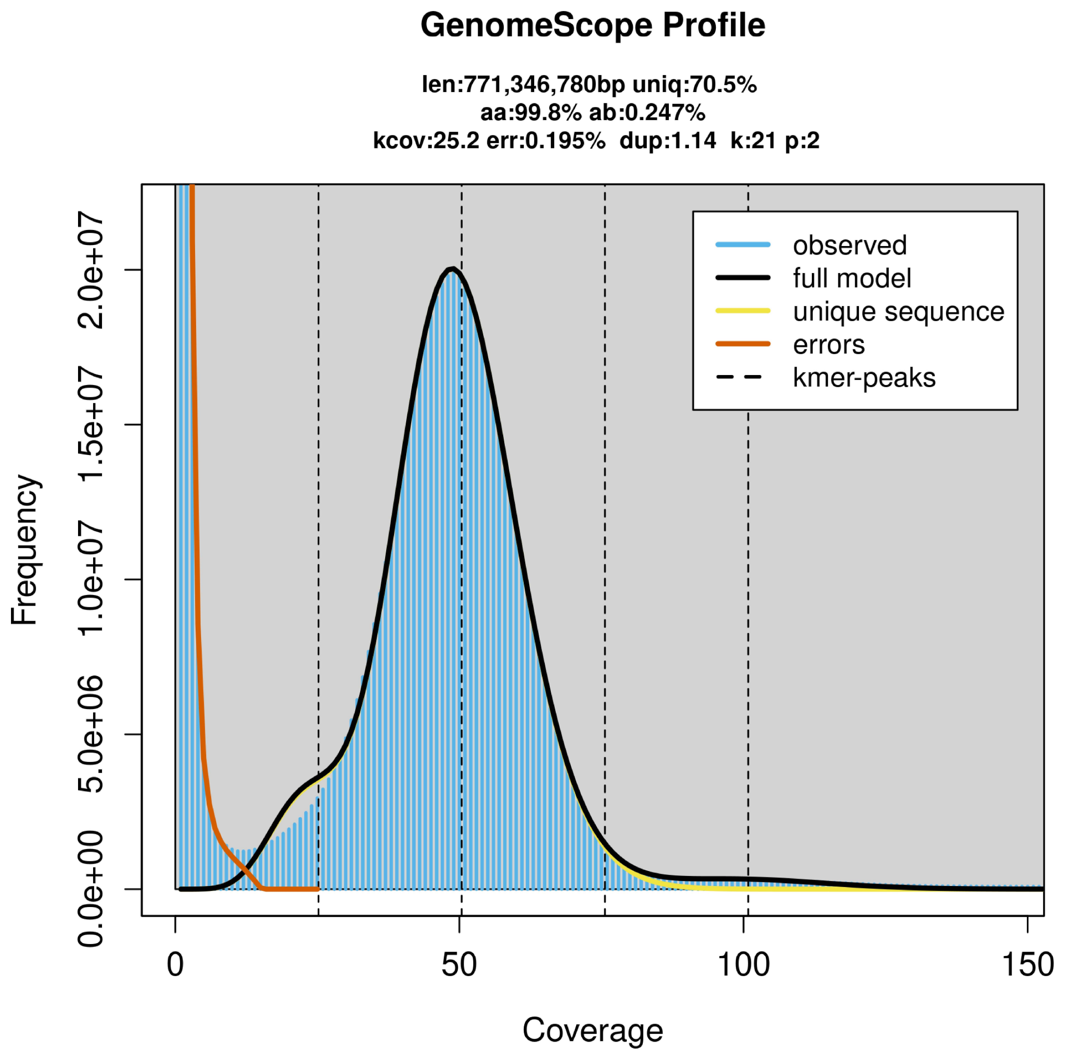

Supplementary Fig. S1. *k*-mer (k=21) analysis for estimating the genome size of a XX *S. lanzhouensis*.

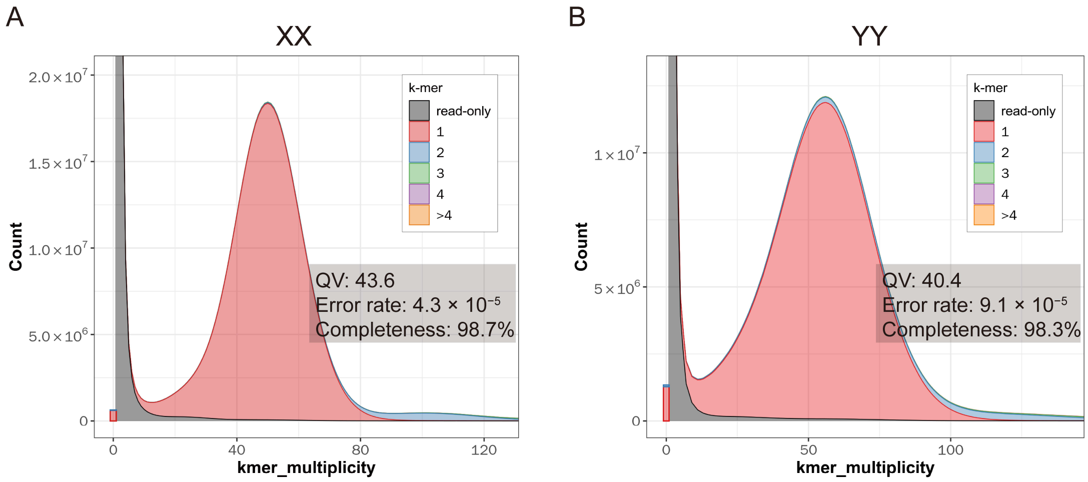

Supplementary Fig. S2. Merqury spectrum plots for *k*-mer-based quality value (QV) and completeness evaluation of XX (A) and YY (B) genome assembly.

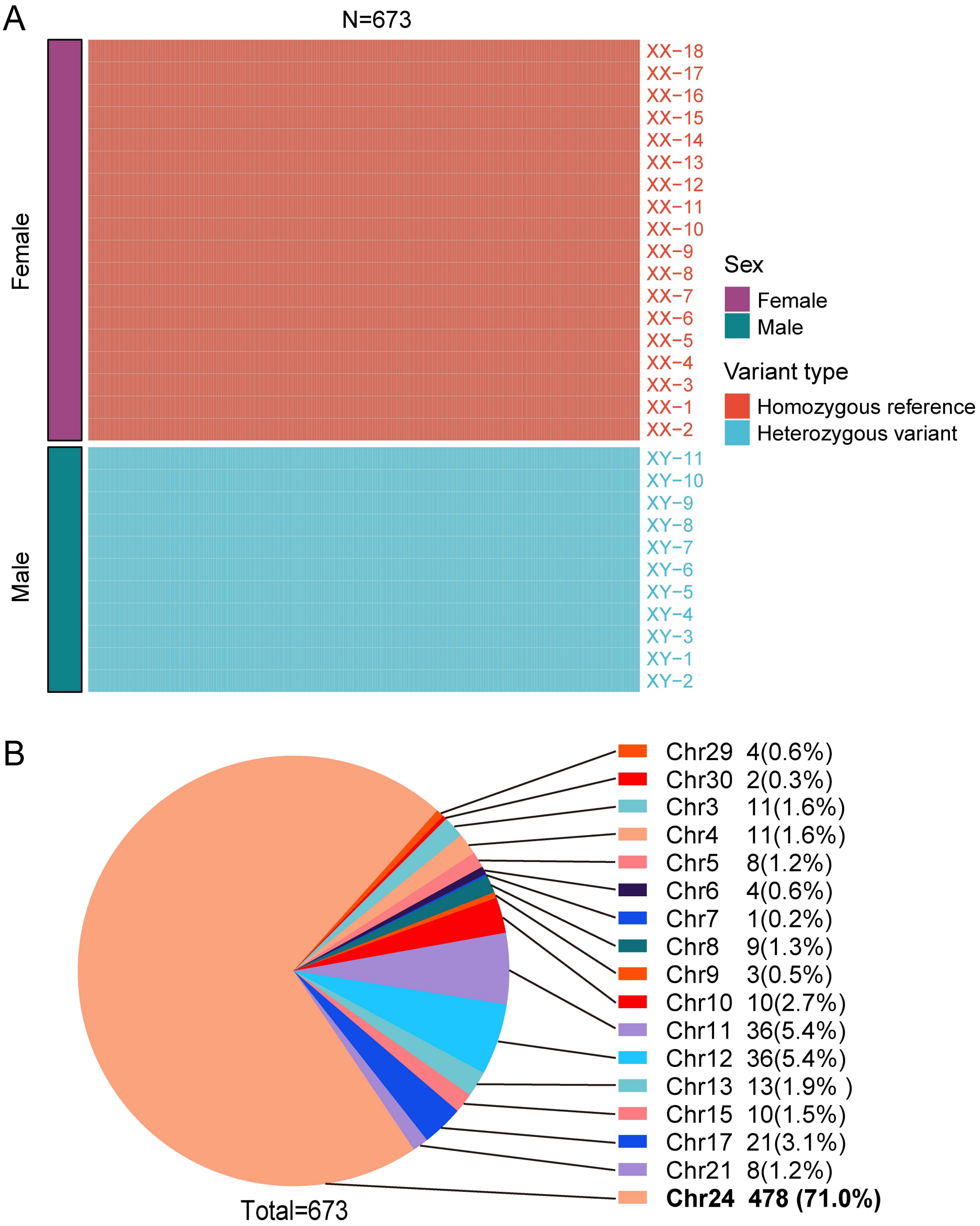

Supplementary Fig. S3. The variant types and chromosomal distribution of male-specific SNPs. A) The variant types of 673 male-specific SNPs between 18 XX female and 11 XY male individuals. Each row corresponds to one individual, and each column corresponds to a SNP. B) Chromosomal distribution of the 673 male-specific SNPs. The chromosome number, the number of SNPs on this chromosome, and the percentage of SNPs on this chromosome are displayed on the right-hand side.

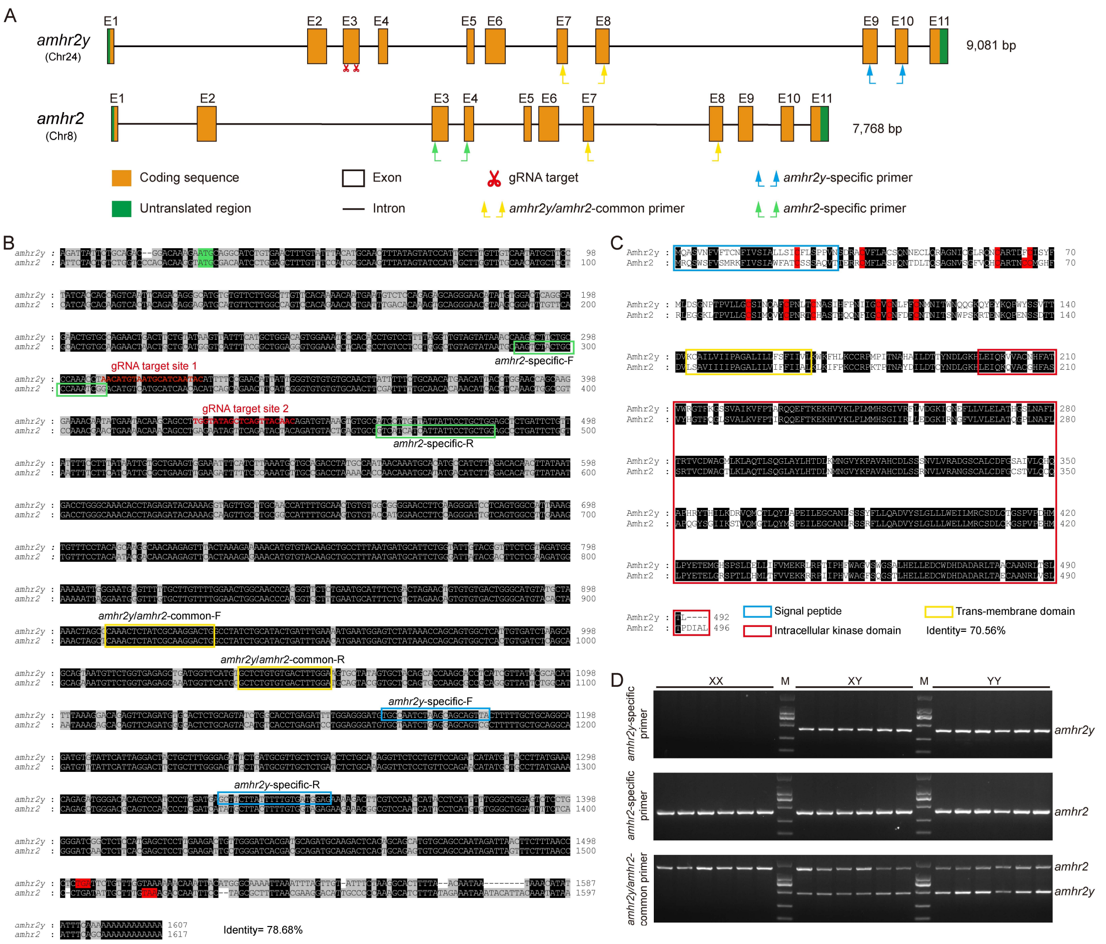

Supplementary Fig. S4. Sequence comparison of *amhr2y* and *amhr2*.

A) Gene structure of *amhr2y* and *amhr2*. B) Sequence alignment of full-length cDNA of *amhr2* and *amhr2y.* Primers were boxed in multicolor. Initiation and termination codons were highlighted with green and red background, respectively. The sequences of knockout target sites were marked in red. C) Alignment of Amhr2y and Amhr2 deduced protein sequences. The conserved cysteines are highlighted with red background. D) PCR detection of *amhr2y*-specific, *amhr2*-specific and *amhr2y*/*amhr2*-common primers in XX, XY and YY individuals.

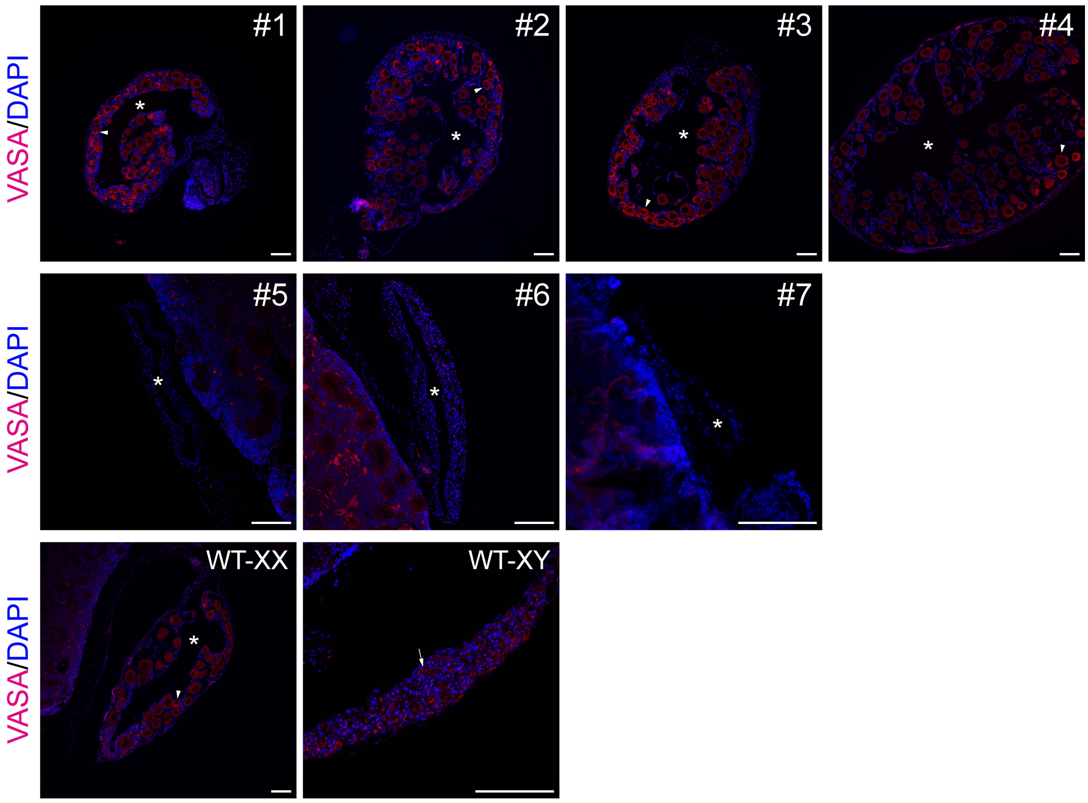

Supplementary Fig. S5. Immunofluorescence of Vasa in seven *amhr2y* knockout individuals and wild type females and males at 50 dah. Red fluorescence was immunostained by anti-Vasa antibody, and blue fluorescence was stained by DAPI. Arrowhead and arrow indicate female and male germ cells, respectively. The asterisk indicates ovarian cavity. Scale bars are 100 µm.

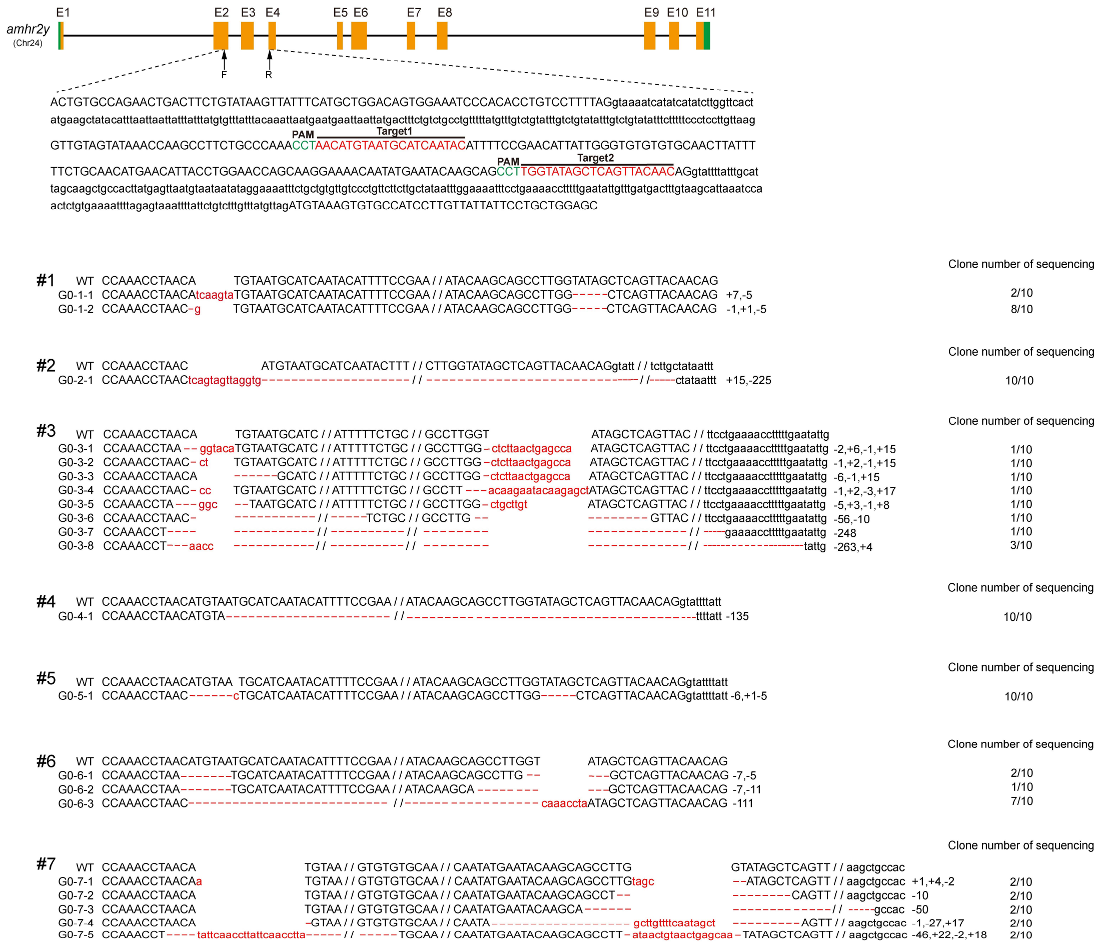

Supplementary Fig. S6. Genotypes in seven *amhr2y* knockout individuals. A total of ten clones were sequenced for each individual, and clone number of each mutation type are listed in right side.

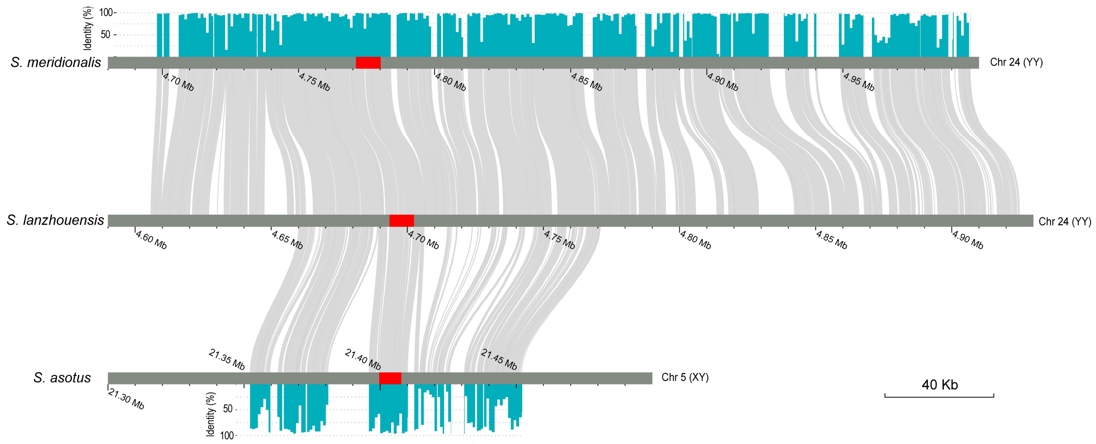

Supplementary Fig. S7. Synteny and identity of conserved blocks among MSYs of three *Silurus* species.

Synteny relationships of syntenic are shown in gray. The identities of the conserved regions between *S. meridionalis* and *S. lanzhouensis* and between *S. asotus* and *S. lanzhouensis* are shown in green. The *amhr2y* gene was highlighted in red.

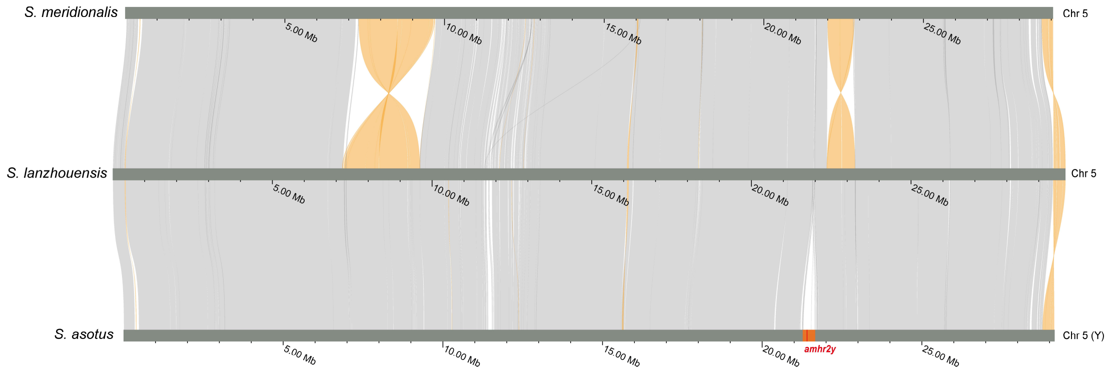

Supplementary Fig. S8. Synteny relationship of Chr5 among three *Silurus* fishes. Synteny relationships of syntenic and reverse regions are shown in gray and yellow, respectively. The MSY and *amhr2y* gene of *S. asotus* were highlighted in orange and red, respectively.

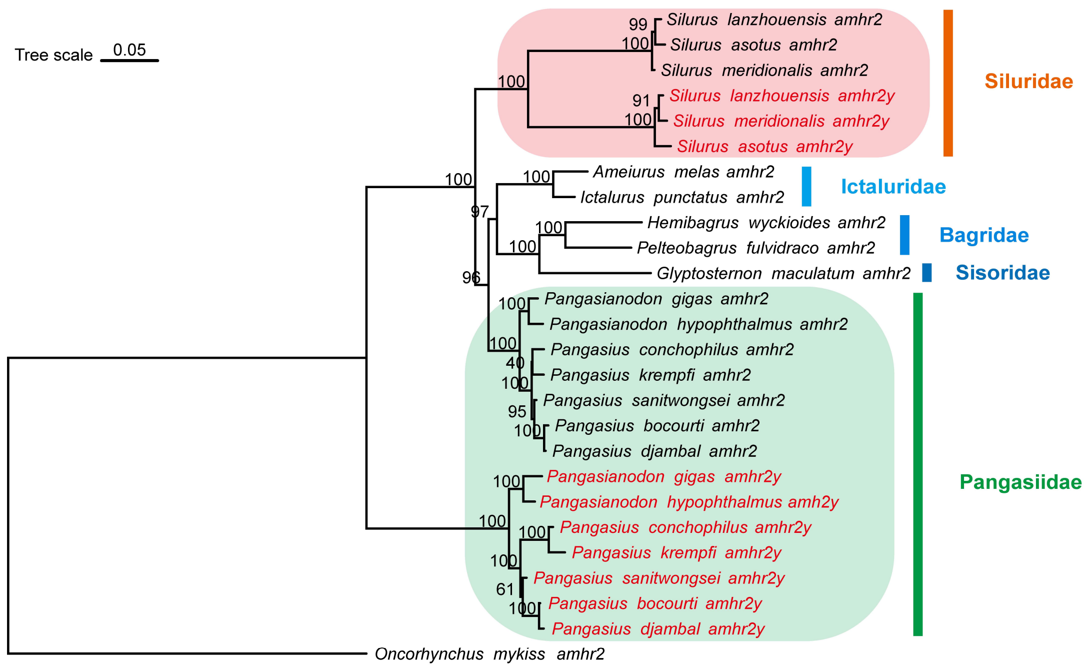

Supplementary Fig. S9. Phylogenetic tree of *amhr2*/*amhr2y* coding sequences in 15 teleosts. *Oncorhynchus mykiss amhr2* is used as an outgroup. The *amhr2y* branches are highlight in red color. Clades of Siluridae and Pangasiidae are indicated in pink and green, respectively.

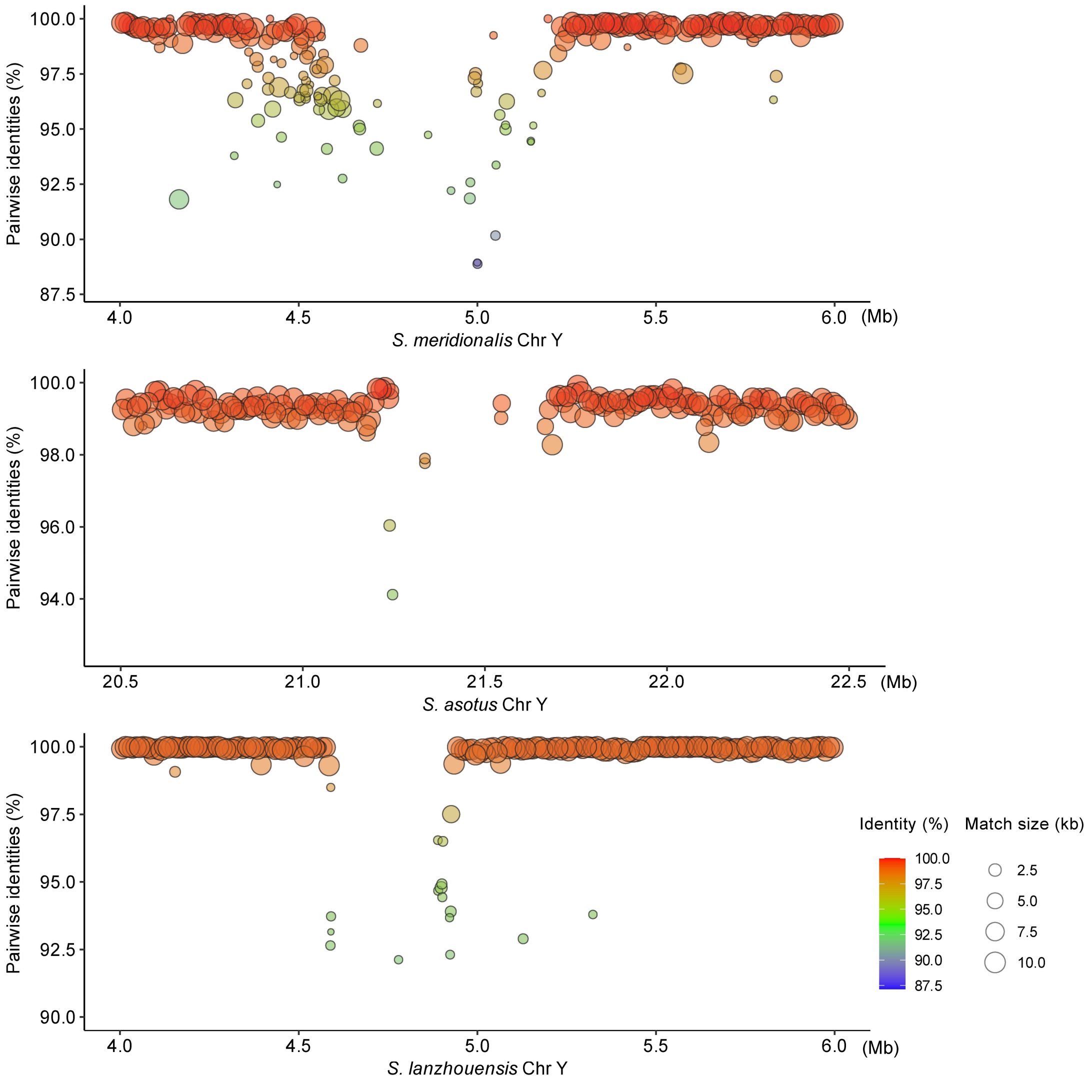

Supplementary Fig. S10. Sequence divergence between sex chromosomes around the sex-specific region in three *Silurus* fishes. Each dot represents a 10-kb sliding window along the Y chromosome. The size and color represent the length and identity of alignments between X and Y chromosome.

Supplementary Table S1. Genomic sequencing data for XX female and YY male *S. lanzhouensis*.

Library type	Platform	Library size	Data size (Gb)/depth (×)	
			XX female	YY male
Long reads	PacBio Sequel II	20-30 kb	132.95 Gb/172×	138.69 Gb/179×
Short reads	Illumina HiSeq X-Ten	350 bp	47.09 Gb/61×	50.79 Gb/65×
Hi-C	Illumina HiSeq X-Ten	350 bp	153.65 Gb/198×	91.96 Gb/118×

Supplementary Table S2. Summary of XX and YY genome assemblies for XX female and YY male *S. lanzhouensis*.

	XX female	YY male
Sequence number (contig)	430	380
Genome size (Mb)	774.75	776.88
Chromosome length (Mb)	773.14 (99.8%)	776.18 (99.9%)
Contig N50 (bp)	6.70	7.12
Scaffold N50 (bp)	28.89	29.43
Complete BUSCOs (%)	97.0	96.6

Supplementary Table S3. Summary of annotation in XX and YY *S. lanzhouensis*.

Assembly	Gene number	Average gene length (bp)	Average CDS length (bp)	Average exons per transcript	Average exon length (bp)	Average intron length (bp)	Repeat of genome (%)
XX	24,836	17,701	1,667	11.0	230	1,387	41.93
YY	24,948	17,685	1,669	11.8	230	1,385	41.58

Supplementary Table S4. Repeat annotation of XX and YY *S. lanzhouensis*.

Assembly	Repeat Class	Repeat Size (bp)	% of genome
XX	Retrotransposons	114,554,706	14.79
	DNA transposons	153,118,841	19.76
	Helitrons	3,018,698	0.39
	Unclassified	43,040,504	5.56
	Simple repeats	11,105,309	1.43
	Total	324,838,058	41.93
YY	Retrotransposons	116484127	14.99
	DNA transposons	153228803	19.72
	Helitrons	2104159	0.27
	Unclassified	41545880	5.35
	Simple repeats	9729078	1.25
	Total	323092047	41.58

Supplementary Table S5. Genome assembles of each chromosome in XX female and YY male *S. lanzhouensis*.

Chromosome ID	XX female		YY male	
	GC content	Length (bp)	GC content	Length (bp)
Chr1	38.90	39,429,733	38.91	40,214,900
Chr2	38.61	37,466,789	38.58	36,729,465
Chr3	38.71	36,419,518	38.74	36,294,112
Chr4	38.81	35,471,113	38.82	36,559,183
Chr5	39.06	34,294,993	39.01	34,484,024
Chr6	38.52	30,772,865	38.56	32,036,961
Chr7	38.91	30,665,606	38.87	31,153,908
Chr8	38.90	30,639,380	38.96	31,134,053
Chr9	38.53	29,836,738	38.51	30,376,046
Chr10	38.96	29,769,092	38.99	30,335,044
Chr11	38.85	29,364,065	38.81	29,611,100
Chr12	38.85	28,889,810	38.85	29,429,150
Chr13	38.82	27,655,847	38.80	27,645,469
Chr14	39.01	27,341,650	38.92	22,390,201
Chr15	38.44	25,942,030	38.43	25,561,456
Chr16	39.06	25,216,937	39.10	26,708,360
Chr17	38.60	24,882,576	38.62	24,456,153
Chr18	38.85	23,987,034	38.86	24,356,124
Chr19	39.14	21,903,936	39.17	22,153,507
Chr20	38.88	21,364,121	39.02	21,723,780
Chr21	39.18	20,846,610	39.07	20,466,399
Chr22	39.28	20,663,463	39.25	20,055,092
Chr23	38.72	20,441,411	38.69	20,343,544
Chr24	38.96	20,232,516	38.93	20,806,263
Chr25	38.65	19,908,025	38.62	19,898,230
Chr26	39.12	18,505,739	39.10	18,468,888
Chr27	39.02	17,806,857	39.10	18,005,945
Chr28	39.56	17,456,887	39.50	18,207,112
Chr29	39.57	13,486,085	39.63	13,960,801
Chr30	39.91	12,478,200	39.77	12,609,841

Supplementary Table S6. Information of 673 male-specific SNPs.

(Excel table)

Supplementary Table S7. The exon features of *nup133y* in *S. meridionalis*.

Exon	Length of *nup133y*/*nup133* (bp)	Start	End	Insertion (nt)	Deletion (nt)	Point mutation (nt)	Synonymous mutation (nt)	Non-synonymous mutation (nt)	Nonsense mutation (nt)
*Sm_nup133y*_E1-1	76/128	5,086,924	5,086,999	0	9	1	0	1	0
*Sm_nup133y*_E4-1	105/105	5,215,420	5,215,524	0	0	7	2	5	0
*Sm_nup133y*_E4-2	61/105	4,834,944	4,834,884	0	0	2	1	1	0
*Sm_nup133y*_E5-1	118/132	4,834,719	4,834,602	1	2	11	2	9	2
*Sm_nup133y*_E5-2	54/132	5,215,926	5,215,979	0	0	1	0	1	0
*Sm_nup133y*_E6-1	121/171	4,834,441	4,834,321	0	0	9	2	7	0
*Sm_nup133y*_E6-2	117/171	5,215,979	5,216,095	0	0	10	3	7	0
*Sm_nup133y*_E8-1	71/71	4,834,185	4,834,115	0	0	2	1	1	0
*Sm_nup133y*_E8-2	68/71	5,216,222	5,216,289	0	0	2	0	2	1
*Sm_nup133y*_E9-1	148/148	5,216,795	5,216,942	0	0	7	2	5	2
*Sm_nup133y*_E9-2	122/148	4,833,597	4,833,476	0	11	9	3	6	2
*Sm_nup133y*_E10-1	150/151	5,217,392	5,217,541	0	0	7	4	3	0
*Sm_nup133y*_E10-2	151/151	4,833,247	4,833,097	0	0	16	9	7	0
*Sm_nup133y*_E11-1	163/158	5,217,605	5,217,767	5	0	9	3	6	0
*Sm_nup133y*_E11-2	98/158	4,832,994	4,832,897	0	2	6	2	4	0
*Sm_nup133y*_E13-1	151/164	5,217,872	5,218,022	0	12	15	5	10	0

*Sm_nup133y*_E14-1	48/95	5,218,148	5,218,195	0	0	1	1	0	0
*Sm_nup133y*_E15-1	106/225	5,218,194	5,218,299	0	0	7	4	3	0
*Sm_nup133y*_E16-1	123/123	5,218,899	5,219,021	0	0	9	6	3	0
*Sm_nup133y*_E18-1	239/252	4,832,052	4,831,814	6	0	27	10	17	0
*Sm_nup133y*_E18-2	170/252	5,219,791	5,219,960	0	1	17	6	11	0
*Sm_nup133y*_E18-3	49/252	5,219,967	5,220,015	0	0	4	1	3	0
*Sm_nup133y*_E19-1	134/134	4,831,651	4,831,518	0	0	3	0	3	1
*Sm_nup133y*_E19-2	107/134	5,220,214	5,220,320	0	0	4	2	2	0
*Sm_nup133y*_E20-1	114/159	4,830,746	4,830,633	0	0	7	3	4	0
*Sm_nup133y*_E21-1	135/136	4,902,672	4,902,538	0	1	12	7	5	0
*Sm_nup133y*_E21-2	64/136	4,907,358	4,907,421	0	0	7	3	4	0
*Sm_nup133y*_E24-1	50/74	4,907,677	4,907,726	0	0	7	1	6	0
*Sm_nup133y*_E25-1	87/89	5,222,401	5,222,487	0	0	10	4	6	0
*Sm_nup133y*_E25-2	54/89	4,908,242	4,908,295	0	0	3	1	2	0
*Sm_nup133y*_E25-3	65/89	5,012,717	5,012,653	0	0	8	3	5	0
*Sm_nup133y*_E26-1	138/137	5,222,589	5,222,726	1	0	10	6	4	1
*Sm_nup133y*_E26-2	137/137	4,909,271	4,909,407	0	0	17	6	11	2
*Sm_nup133y*_E26-3	137/137	4,889,613	4,889,477	0	0	18	7	11	2
*Sm_nup133y*_E26-4	64/137	5,012,557	5,012,494	0	0	10	4	6	2

Supplementary Table S8. The exon features of *acta2y* in *S. meridionalis*.

Exon	Length of *acta2y*/*acta2* (bp)	Start	End	Insertion (nt)	Deletion (nt)	Point mutation (nt)	Synonymous mutation (nt)	Non-synonymous mutation (nt)	Nonsense mutation (nt)
*Sm_acta2y*_E2-1	141/142	5,129,706	5,129,846	0	1	10	3	7	0
*Sm_acta2y*_E2-2	35/142	4,911,785	4,911,819	0	0	2	0	2	0
*Sm_acta2y*_E3-1	300/325	5,061,707	5,061,408	1	7	15	7	8	0
*Sm_acta2y*_E3-2	216/325	4,862,665	4,862,880	0	7	9	3	6	0
*Sm_acta2y*_E3-3	87/325	5,131,095	5,131,181	0	0	2	1	1	0
*Sm_acta2y*_E3-4	26/325	4,862,899	4,862,924	0	0	0	0	0	0
*Sm_acta2y*_E3-5	30/325	5,130,881	5,130,910	0	0	1	0	1	0
*Sm_acta2y*_E4-1	117/162	4,981,170	4,981,054	0	0	3	1	2	0
*Sm_acta2y*_E4-2	118/162	5,076,625	5,076,508	0	0	5	2	3	0
*Sm_acta2y*_E4-3	115/162	5,146,116	5,146,230	0	0	5	2	3	0
*Sm_acta2y*_E4-4	118/162	4,692,654	4,692,771	0	0	7	1	6	0
*Sm_acta2y*_E4-5	70/162	5,060,595	5,060,526	0	0	1	0	1	0
*Sm_acta2y*_E4-6	70/162	5,131,476	5,131,545	0	0	1	0	1	0
*Sm_acta2y*_E4-7	67/162	5,060,659	5,060,593	0	0	1	0	1	0
*Sm_acta2y*_E4-8	76/162	4,863,750	4,863,825	0	0	5	1	4	0
*Sm_acta2y*_E4-9	58/162	4,863,689	4,863,746	0	0	2	2	0	0

*Sm_acta2y*_E4-10	63/162	5,131,412	5,131,474	0	0	4	1	3	0
*Sm_acta2y*_E5-1	196/192	5,131,633	5,131,828	4	0	2	2	5	0
*Sm_acta2y*_E5-2	125/192	4,863,912	4,864,036	3	0	1	1	2	0
*Sm_acta2y*_E5-3	129/192	4,670,484	4,670,356	0	7	1	3	4	0
*Sm_acta2y*_E5-4	73/192	5,076,418	5,076,346	0	0	0	0	2	0
*Sm_acta2y*_E5-5	105/192	5,060,421	5,060,317	3	0	1	2	5	0
*Sm_acta2y*_E5-6	73/192	4,980,964	4,980,892	0	0	0	2	0	0
*Sm_acta2y*_E5-7	57/192	4,692,860	4,692,916	0	0	0	1	4	0
*Sm_acta2y*_E5-8	35/192	4,864,041	4,864,075	0	0	0	0	0	0
*Sm_acta2y*_E6-1	177/182	4,864,157	4,864,333	0	0	11	4	7	0
*Sm_acta2y*_E6-2	127/182	5,131,941	5,132,067	0	0	0	0	0	0
*Sm_acta2y*_E6-3	127/182	4,912,920	4,913,046	0	0	6	2	4	0
*Sm_acta2y*_E6-4	33/182	4,670,231	4,670,199	0	0	2	1	1	0
*Sm_acta2y*_E6-5	26/182	4,670,268	4,670,243	0	0	1	0	1	0
*Sm_acta2y*_E7-1	144/144	5,192,898	5,193,041	0	0	0	0	0	0
*Sm_acta2y*_E7-2	144/144	4,913,796	4,913,939	0	0	3	0	3	0
*Sm_acta2y*_E7-3	53/144	4,866,695	4,866,747	0	0	4	0	4	0

Supplementary Table S9. The exon features of *kif16by* in *S. meridionalis*.

Exon	Length of *kif16by*/*kif16b* (bp)	Start	End	Insertion (nt)	Deletion (nt)	Point mutation (nt)	Synonymous mutation (nt)	Non-synonymous mutation (nt)	Nonsense mutation (nt)
*Sm_kif16by*_E4-1	115/117	5,213,368	5,213,254	0	2	7	6	1	1
*Sm_kif16by*_E4-2	106/117	4,836,437	4,836,542	0	0	10	5	5	0
*Sm_kif16by*_E5-1	95/98	5,205,406	5,205,312	0	0	5	2	3	0
*Sm_kif16by*_E5-2	48/98	4,837,384	4,837,431	0	0	5	0	5	0
*Sm_kif16by*_E6-1	110/110	5,204,291	5,204,182	0	0	3	3	0	0
*Sm_kif16by*_E6-2	104/110	4,838,500	4,838,603	0	4	10	5	5	0
*Sm_kif16by*_E7-1	141/143	5,204,098	5,203,958	0	2	5	2	3	0
*Sm_kif16by*_E7-2	139/143	4,838,691	4,838,829	0	0	7	6	1	0
*Sm_kif16by*_E24-1	103/159	5,065,413	5,065,515	0	0	5	1	5	0
*Sm_kif16by*_E24-2	39/159	5,065,375	5,065,413	0	0	2	1	1	0
*Sm_kif16by*_E26-1	42/45	5,066,164	5,066,205	0	0	0	0	0	0
*Sm_kif16by*_E27-1	116/123	5,066,293	5,066,408	0	7	10	4	6	0

Supplementary Table S10. The exon features of *nup133y* in *S. lanzhouensis*.

Exon	Length of *nup133y*/*nup133* (bp)	Start	End	Insertion (nt)	Deletion (nt)	Point mutation (nt)	Synonymous mutation (nt)	Non-synonymous mutation (nt)	Nonsense mutation (nt)
*Sl_nup133y*_E4-1	64/105	4,751,682	4,751,619	0	0	2	1	1	0
*Sl_nup133y*_E5-1	131/132	4,751,454	4,751,324	2	0	9	3	6	0
*Sl_nup133y*_E6-1	122/171	4,751,176	4,751,055	0	0	11	4	7	0
*Sl_nup133y*_E8-1	71/71	4,750,913	4,750,843	0	0	0	0	0	0
*Sl_nup133y*_E9-1	122/148	4,750,277	4,750,156	0	11	8	4	4	0
*Sl_nup133y*_E10-1	151/151	4,749,907	4,749,757	0	0	14	8	6	0
*Sl_nup133y*_E11-1	98/158	4,749,652	4,749,555	0	2	6	3	3	0
*Sl_nup133y*_E17-1	25/100	4,749,355	4,749,331	0	0	1	0	1	0
*Sl_nup133y*_E18-1	264/252	4,748,743	4,748,480	12	0	27	11	16	1
*Sl_nup133y*_E19-1	132/134	4,748,316	4,748,185	0	0	4	0	4	1
*Sl_nup133y*_E20-1	114/159	4,747,584	4,747,471	0	0	8	3	5	0
*Sl_nup133y*_E21-1	136/136	4,827,868	4,827,733	0	0	10	8	2	0
*Sl_nup133y*_E21-2	64/136	4,845,159	4,845,222	0	0	7	4	3	0
*Sl_nup133y*_E22-1	64/119	4,845,366	4,845,429	0	0	2	1	1	0
*Sl_nup133y*_E22-2	31/119	4,827,657	4,827,627	0	0	0	0	0	0
*Sl_nup133y*_E24-1	50/74	4,845,480	4,845,529	0	0	5	1	4	0

*Sl_nup133y*_E25-1	54/89	4,846,057	4,846,110	0	0	3	1	2	0
*Sl_nup133y*_E25-2	87/89	4,815,429	4,815,343	0	0	15	2	13	0
*Sl_nup133y*_E26-1	137/137	4,815,241	4,815,105	0	0	20	7	13	3

Supplementary Table S11. The exon features of *acta2y* in *S. lanzhouensis*.

Exon	Length of *acta2y*/*acta2* (bp)	Start	End	Insertion (nt)	Deletion (nt)	Point mutation (nt)	Synonymous mutation (nt)	Non-synonymous mutation (nt)	Nonsense mutation (nt)
*Sl_acta2y*_E2-1	142/142	4,848,986	4,849,127	0	0	12	2	10	0
*Sl_acta2y*_E3-1	216/325	4,778,791	4,779,006	0	7	8	4	4	0
*Sl_acta2y*_E4-1	162/162	4,779,826	4,779,987	0	0	6	3	3	0
*Sl_acta2y*_E4-2	118/162	4,908,515	4,908,398	0	0	5	1	4	0
*Sl_acta2y*_E5-1	125/192	4,7800,74	4,780,198	3	0	2	0	2	0
*Sl_acta2y*_E5-2	73/192	4,908,346	4,908,274	0	0	3	2	1	0
*Sl_acta2y*_E5-3	35/192	4,780,203	4,780,237	0	0	0	0	0	0
*Sl_acta2y*_E6-1	177/182	4,780,319	4,780,495	0	0	6	3	3	0
*Sl_acta2y*_E6-2	127/182	4,850,229	4,850,355	0	0	6	2	4	0
*Sl_acta2y*_E7-1	144/144	4,851,103	4,851,246	0	0	3	0	3	0
*Sl_acta2y*_E7-2	53/144	4,782,850	4,782,902	0	0	2	0	2	0

Supplementary Table S12. The exon features of *kif16by* in *S. lanzhouensis*.

Exon	Length of *kif16by*/*kif16b* (bp)	Start	End	Insertion (nt)	Deletion (nt)	Point mutation (nt)	Synonymous mutation (nt)	Non-synonymous mutation (nt)	Nonsense mutation (nt)
*Sl_kif16by*_E4-1	106/117	4,755,346	4,755,451	0	0	9	5	4	0
*Sl_kif16by*_E5-1	52/98	4,756,273	4,756,324	0	0	4	0	4	0
*Sl_kif16by*_E6-1	108/110	4,757,436	4,757,543	0	0	8	4	4	0
*Sl_kif16by*_E7-1	139/143	4,757,631	4,757,769	0	0	5	4	1	0

Supplementary Table S13. The exon features of *nup133y* in *S. asotus*.

Exon	Length of *nup133y*/*nup133* (bp)	Start	End	Insertion (nt)	Deletion (nt)	Point mutation (nt)	Synonymous mutation (nt)	Non-synonymous mutation (nt)	Nonsense mutation (nt)
*Sa_nup133y*_E4-1	57/105	21,437,674	21,437,618	0	0	1	0	1	0
*Sa_nup133y*_E5-1	129/132	21,437,453	21,437,325	0	0	13	4	9	1
*Sa_nup133y*_E6-1	116/171	21,437,178	21,437,063	1	0	11	1	10	0
*Sa_nup133y*_E8-1	64/71	21,436,941	21,436,878	0	0	2	1	1	1
*Sa_nup133y*_E9-1	122/148	21,436,306	21,436,185	0	11	12	4	8	0
*Sa_nup133y*_E10-1	130/151	21,435,884	21,435,755	0	0	10	5	5	0
*Sa_nup133y*_E11-1	90/158	21,435,648	21,435,559	0	2	7	4	3	0
*Sa_nup133y*_E12-1	86/89	21,435,382	21,435,297	0	1	7	3	4	0
*Sa_nup133y*_E13-1	65/164	21,435,192	21,435,128	0	0	6	1	5	0
*Sa_nup133y*_E17-1	37/100	21,434,657	21,434,621	0	0	4	1	3	0
*Sa_nup133y*_E18-1	241/252	21,434,039	21,433,799	6	0	28	14	14	0
*Sa_nup133y*_E19-1	132/134	21,433,662	21,433,531	0	0	6	2	4	1
*Sa_nup133y*_E20-1	122/159	21,432,916	21,432,795	0	0	13	3	10	1

Supplementary Table S14. The exon features of *kif16by* in *S. asotus*.

Exon	Length of *kif16by*/*kif16b* (bp)	Start	End	Insertion (nt)	Deletion (nt)	Point mutation (nt)	Synonymous mutation (nt)	Non-synonymous mutation (nt)	Nonsense mutation (nt)
*Sa_kif16by*_E4-1	106/117	21,439,847	21,439,952	0	0	7	4	3	0
*Sa_kif16by*_E5-1	50/98	21,440,820	21,440,869	0	0	2	0	2	0
*Sa_kif16by*_E6-1	104/110	21,441,933	21,442,036	0	4	7	5	2	1
*Sa_kif16by*_E7-1	131/143	21,442,124	21,442,254	0	0	11	5	6	0

Supplementary Table S15. The purged primary assemblies of XX individual using different assemblers.

Assemblers	Version	Total length (Mb)	Number of contigs	Contig N50 (Mb)
Falcon	1.3.0	774.69	419	6,71
Canu	2.2	782.83	617	6.72
wtdbg2	2.5	751.08	713	8.94
Flye	2.9.2	764.40	344	7.62
MECAT2	20190314	758.77	672	6.80

Supplementary Table S16. Primers used in this study.

Primer name	Primer sequence (5′−3′)	Usage
Chr24-probe-1F	TGTAACTTAATCACTGCTAATCCTC	*In-situ* hybridization
Chr24-probe-1R	CCGTCTGAGAACAGCCAAG	*In-situ* hybridization
Chr24-probe-2F	CTCACTGGCGGCTGGAAG	*In-situ* hybridization
Chr24-probe-2R	TGCCTTACTCTGGTGGACTATG	*In-situ* hybridization
Chr24-probe-3F	TCCTTCTAAACAATAGTCGAATCAG	*In-situ* hybridization
Chr24-probe-3R	GCAGCACCAGCATCCATC	*In-situ* hybridization
Chr24-probe-4F	GTGTGCTTGCTGTTAGTTAGAG	*In-situ* hybridization
Chr24-probe-4R	GTGAGATGTAGGTTCCAGGTATC	*In-situ* hybridization
Chr24-probe-5F	CTGTTGTGTTTAGGCTCTTTATTTG	*In-situ* hybridization
Chr24-probe-5R	TTGTGCTGTTTGTTTATTTAACTGG	*In-situ* hybridization
Chr24-probe-6F	TTGTGGCTCTGCTGTTAGTAATC	*In-situ* hybridization
Chr24-probe-6R	TTCCATCCCAAATCTCCCTCTC	*In-situ* hybridization
Chr24-probe-7F	CACACGCACTAATAAGATGGATG	*In-situ* hybridization
Chr24-probe-7R	CACAAATCAGAGCAGTCACAAG	*In-situ* hybridization
Chr24-probe-8F	CTCTGCCACCTCCTGATAATG	*In-situ* hybridization
Chr24-probe-8R	TGGTATTCTCTAACTGGACTTCTG	*In-situ* hybridization
Chr24-probe-9F	AGGGTGTGCCAGATATTCAAATAC	*In-situ* hybridization
Chr24-probe-9R	ACGAGCCTGTAAGGTGTAGATG	*In-situ* hybridization
Chr24-probe-10F	GCTGGAGATTGTACGAGTGTAG	*In-situ* hybridization
Chr24-probe-10R	AATGATGTTTAAGGTTCAGGTTTCC	*In-situ* hybridization
Chr24-probe-11F	CGACTTAGAGCAGAGCGATTAC	*In-situ* hybridization
Chr24-probe-11R	CTGGTCAGGGTCACGGAAG	*In-situ* hybridization
Chr24-probe-12F	TGCGAGTAACGAACTGATAGTAATC	*In-situ* hybridization
Chr24-probe-12R	TGACTGTAACATTGTGACCTTTGG	*In-situ* hybridization
Chr24-probe-13F	GCCAGCCACAGGAAACTTTG	*In-situ* hybridization

Chr24-probe-13R	GGAGCCTCATTGACTTCTTAACAG	*In-situ* hybridization
Chr24-probe-14F	TCCACCTGCCACACCAAC	*In-situ* hybridization
Chr24-probe-14R	CCACAGCACCATACAGTAGAAG	*In-situ* hybridization
Chr24-probe-15F	GTCTATACTGGGAACATTCTGGTC	*In-situ* hybridization
Chr24-probe-15R	GCAACTGTAGTGAAGGCAATAAC	*In-situ* hybridization
Chr24-probe-16F	AAGGCTTTCTGCTGGAGTTTG	*In-situ* hybridization
Chr24-probe-16R	GGCTTGTGAGATGAGTGAGATG	*In-situ* hybridization
Chr24-probe-17F	ATCTGAGCCGTGGATATAAAGTTC	*In-situ* hybridization
Chr24-probe-17R	CCCGATATGAAGCGTTCTGAAG	*In-situ* hybridization
Chr24-probe-18F	TGTCTGTTGTAGGCTGAGGAG	*In-situ* hybridization
Chr24-probe-18R	GCTTGTGAGATGAGTGAGATGG	*In-situ* hybridization
Chr24-probe-19F	GCATCCTCATTACTTGGCACTC	*In-situ* hybridization
Chr24-probe-19R	TTCTTGGCGACTCCCTTCAC	*In-situ* hybridization
Chr24-probe-20F	TGGTGGTGCTGCTCATCTAC	*In-situ* hybridization
Chr24-probe-20R	TGACTCTGGGCTACACAACTC	*In-situ* hybridization
Chr24-probe-21F	GCCATCATCATCATCATCATCATC	*In-situ* hybridization
Chr24-probe-21R	CATCGCTTCTGTGTTTCTGTTC	*In-situ* hybridization
Chr24-probe-22F	AAGCCGAGTGTCCGAGAG	*In-situ* hybridization
Chr24-probe-22R	CTGTGAAGGTTATGAAGGTTGTG	*In-situ* hybridization
amhr2y-probe-1F	GCCTTTCCTGGTGCATTTCTAC	*In-situ* hybridization
amhr2y-probe-1R	TGCTGGTTCCTGCTGGTATC	*In-situ* hybridization
amhr2y-probe-2F	TGTGTGTGTTTGTGTGTGAATTAG	*In-situ* hybridization
amhr2y-probe-2R	CTGTGCTGAACTGCTGGAAG	*In-situ* hybridization
amhr2y-probe-3F	CCCACTAAACCCTGCCACTC	*In-situ* hybridization
amhr2y-probe-3R	CTTGATGTCACCACCCATACTTG	*In-situ* hybridization
amhr2y-probe-4F	TTAGCAATCTGAGCGAACCTTG	*In-situ* hybridization
amhr2y-probe-4R	ACAAGAAGAAGATGGCTGAACTC	*In-situ* hybridization
amhr2y-probe-5F	GTAACGAACAGATCGCATATTTGG	*In-situ* hybridization
amhr2y-probe-5R	CCTCTGTCAGTATGAGACTTAATC<br>C	*In-situ* hybridization

amhr2y-probe-6F	ATTGAATGTAAGATGTCTGATGGTG	*In-situ* hybridization
amhr2y-probe-6R	ACTGTTTGCTTGCTAACTTTGG	*In-situ* hybridization
amhr2y-probe-7F	ATGGTGTGAATGGAAAGAGAAATG	*In-situ* hybridization
amhr2y-probe-7R	GTGGTATTAAGAGGTGGATTAAGTC	*In-situ* hybridization
amhr2y-probe-8F	GCCCTGACCACCAAACAC	*In-situ* hybridization
amhr2y-probe-8R	TACTTCCCACCTCTGATAATACTAC	*In-situ* hybridization
amhr2y-probe-9F	TTTGCCACCTCTAAATATGATTGAG	*In-situ* hybridization
amhr2y-probe-9R	TAATTAACAGCTTCTGACCACTTG	*In-situ* hybridization
amhr2y-probe-10F	TCCTAAAAGACAATGAAATCCTAGC	*In-situ* hybridization
amhr2y-probe-10R	CTGACTGACTGACTGACTGAC	*In-situ* hybridization
amhr2y-probe-11F	TTCGTGATCCGCATGTTTGAC	*In-situ* hybridization
amhr2y-probe-11R	GATTACCAATTCGCTGTTGTTACTC	*In-situ* hybridization
amhr2-qPCR-F	GATACAAAAGCAAGTTGCCTGCGG	qPCR
amhr2-qPCR-R	AGGACCTTGGGTTGCCAGTTCC	qPCR
amhr2y-qPCR-F	GCTCTGTGTGACTTTGGAAG	qPCR
amhr2y-qPCR-R	ATGAGGTATGGTTGGACGAA	qPCR
beta-actin-qPCR-F	GCCCAAGACACCAGGGTGTG	qPCR
beta-actin-qPCR-R	GAAGGTCTCAAACATAATCTGGGTC	qPCR
amh-qPCR-F	CAGTCCAGTCATTGCCAGG	qPCR
amh-qPCR-R	GGTGATAGTCCCGTCTTCTTCG	qPCR
gsdf-qPCR-F	GCAGAAGACAACACACAACCAA	qPCR
gsdf-qPCR-R	TACAGGAAGGGCACGGAGT	qPCR
foxl2-qPCR-F	ACGAGAAGAATAAGAAAGGCTGG	qPCR
foxl2-qPCR-R	AGAGAGGTATCCGTAACTGTCCC	qPCR
cyp19a1a-qPCR-F	GGTTCTCCTGCTCACCGTT	qPCR
cyp19a1a-qPCR-R	GAAGATGATTCCCTGCTCGT	qPCR

amhr2-spe-F	AAGTCTACTGCCCAAATCGG	Genotyping
amhr2-spe-R	CCAGCAGGAATAATGATGATGAC	Genotyping
amhr2y-spe-F	TGCCAATCTAAGCAGCAGTTA	Genotyping
amhr2y-spe-R	CTCCATCACAAAAATAAGAAGC	Genotyping
amhr2/amhr2y-com-F	CAAACTCTATCGCAAGGACTG	Genotyping
amhr2/amhr2y-com-R	TCCAAAGTCACACAGAGC	Genotyping
amhr2-5' RACE GSP	GTACACACTTGCAAAATGGCC	RACE PCR
amhr2-5' RACE GSP Nested	ATGATGATGACGGCACTCAG	RACE PCR
amhr2-3' RACE GSP	GCCGCAGGGTTATTCTGG	RACE PCR
amhr2-3' RACE GSP Nested	TTTGTGGTAGAGAAGAAACGG	RACE PCR
amhr2y-5' RACE GSP	GAGCTCCAGCAGGAATAATAACA	RACE PCR
amhr2y-5' RACE GSP Nested	CACACCCAATAATGTTCGGAAA	RACE PCR
amhr2y-3' RACE GSP	GTGCCAATCTAAGCAGCAGTTA	RACE PCR
amhr2y-3' RACE GSP Nested	GTTGCTCTGACCTCTGCAC	RACE PCR
T7-*amhr2y*-gRNA1	GTAATACGACTCACTATAGTATTGATGCATTACATGTTGTTTTAGAGCTAGAAATAGC	*In vitro* transcription of gRNA
T7-*amhr2y*-gRNA2	GTAATACGACTCACTATAGTTGTAACTGAGCTATACCAGTTTTAGAGCTAGAAATAGC	*In vitro* transcription of gRNA

Supplementary Table S17. Origin of *amhr2*/*amhr2y* sequences used for phylogenetic analysis.

Species	Gene	Source
Pangasianodon hypophthalmus	*amhr2*	https://doi.org/10.15454/M3HYAX
Pangasianodon hypophthalmus	*amhr2y*	
Pangasianodon gigas	*amhr2*	
Pangasianodon gigas	*amhr2y*	
Pangasius sanitwongsei	*amhr2*	
Pangasius sanitwongsei	*amhr2y*	
Pangasius djambal	*amhr2*	
Pangasius djambal	*amhr2y*	
Pangasius bocourti	*amhr2*	
Pangasius bocourti	*amhr2y*	
Pangasius conchophilus	*amhr2*	
Pangasius conchophilus	*amhr2y*	
Pangasius krempfi	*amhr2*	
Pangasius krempfi	*amhr2y*	
Ictalurus punctatus	*amhr2*	XP_017331275.1
Ameiurus melas	*amhr2*	KAF4083677.1
Hemibagrus wyckioides	*amhr2*	KAG7327988.1
Pelteobagrus fulvidraco	*amhr2*	XP_027015428.2
Glyptosternon maculatum	*amhr2*	http://gigadb.org/dataset/100489
Silurus meridionalis	*amhr2*	inferred from genome assembly (GWHBHES00000000, NGDC)
Silurus meridionalis	*amhr2y*	
Silurus lanzhouensis	*amhr2*	Genome annotation in this study
Silurus lanzhouensis	*amhr2y*	
Silurus asotus	*amhr2*	inferred from genome assembly (GWHAZIF00000000, NGDC)
Silurus asotus	*amhr2y*	
Oncorhynchus mykiss	*amhr2*	XP_036806764.1
